# Supplementary material for: Inflammatory Responses and Barrier Function of Endothelial Cells Derived from Human Induced Pluripotent Stem Cells
Source: Stem Cell Reports. 2018 Apr 12;10(5):1642–56. doi: 10.1016/j.stemcr.2018.03.012 (PMC5995303; doi:10.1016/j.stemcr.2018.03.012)
Supplement: Document S1. Supplemental Experimental Procedures, Figures S1–S7, and Table S1 [file mmc1.pdf]

Stem Cell Reports, Volume *10*

Supplemental Information

Inflammatory Responses and Barrier Function of Endothelial Cells Derived from Human Induced Pluripotent Stem Cells

Oleh V. Halaidych, Christian Freund, Francijna van den Hil, Daniela C.F. Salvatori, Mara Riminucci, Christine L. Mummery, and Valeria V. Orlova

Supplemental Figures and Legends

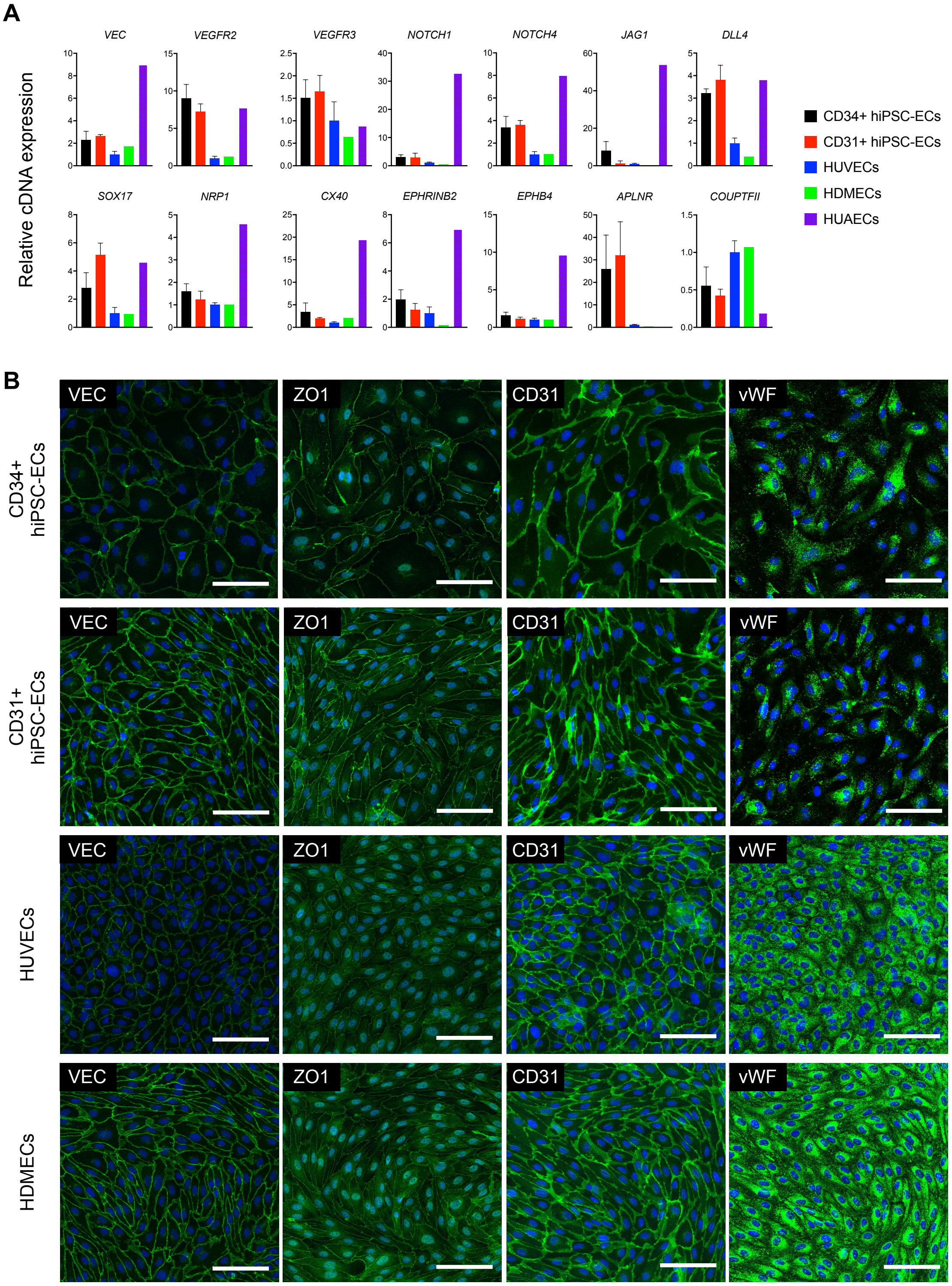

Figure S1. Related to Figure 1. Comparison of hiPSC-derived and primary ECs. (A) Gene expression analysis of expression of arterial and venous markers in isolated CD34+ and CD31+ hiPSC-ECs at passage 2 (P2) and primary ECs (HUVECs, HDMECs and HUAECs). Average values for three batches of CD31+ and CD34+ hiPSC-ECs, HUVECs from three batches (two donors, and two independent batches for one of the donors), HDMECs and HUAECs from a single donor are shown. Error bars are ±SD. (B) Immunofluorescent analysis of EC markers VEC, ZO1, CD31 and vWF on isolated CD34+ and CD31+ hiPSC-ECs (P2) and primary ECs (HUVECs and HDMECs)(P4-P5). Scale bar 100μm.

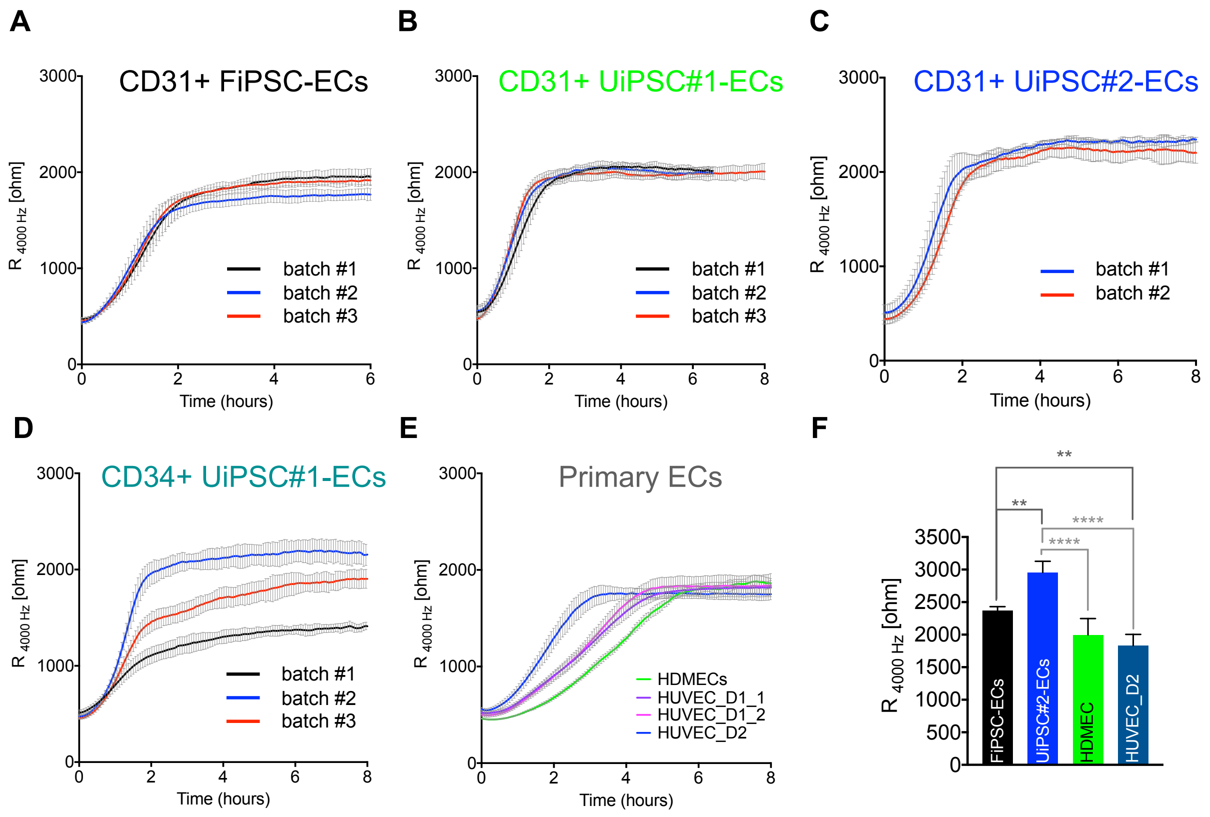

Figure S2. Related to Figure 2. Barrier properties of hiPSC-derived and primary ECs.
(A-E) Absolute resistance of independent batches of CD31+ and CD34+ hiPSC-derived and primary ECs: CD31+ FiPSC-ECs (A), CD31+ UiPSC#1-ECs (B), CD31+ UiPSC#2-ECs (C), CD34+ UiPSC#1-ECs (D), HDMECs and HUVECs (derived from two independent donors D1 and D2, and two independent isolations per donor D1_1 and D1_2) (E). Error bars are shown as ±SD of three to four independent wells. **(F)** Quantification of absolute resistance of CD31+ hiPSC-ECs and primary ECs (HDMECs and HUVECs from donor D2) from three independent biological experiments of the EC monolayer in EGM-2. Error bars are shown as ±SD of three independent biological experiments.

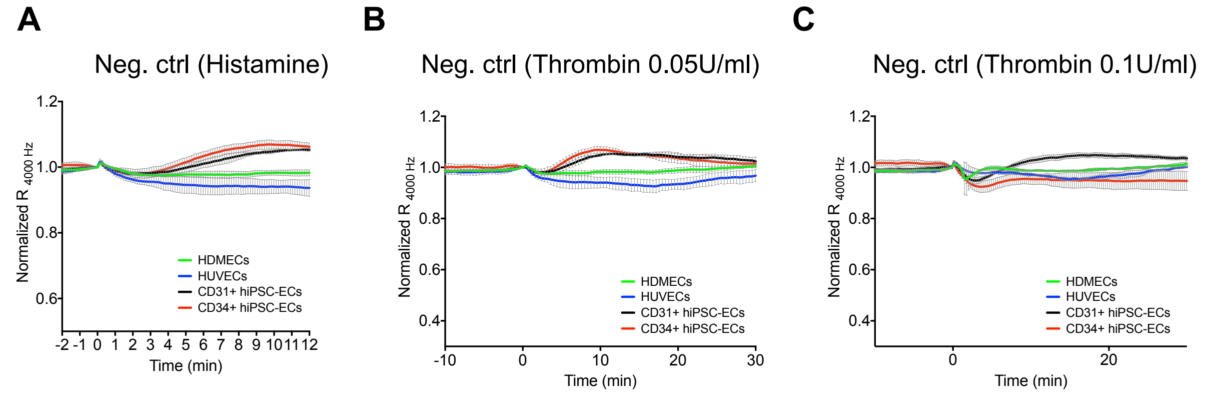

Figure S3. Related for Figure 3. Comparative assessment of barrier disruption in primary and hiPSC-derived ECs upon control (compound-free) treatment.
(A-C) Changes in normalized resistance at 4000 Hz of the endothelial monolayer upon control stimulation with equal volume of medium without the compound is shown. Normalized resistance is shown as a representative plot of one independent biological experiment. Error bars are shown ±SD of three to four independent wells.

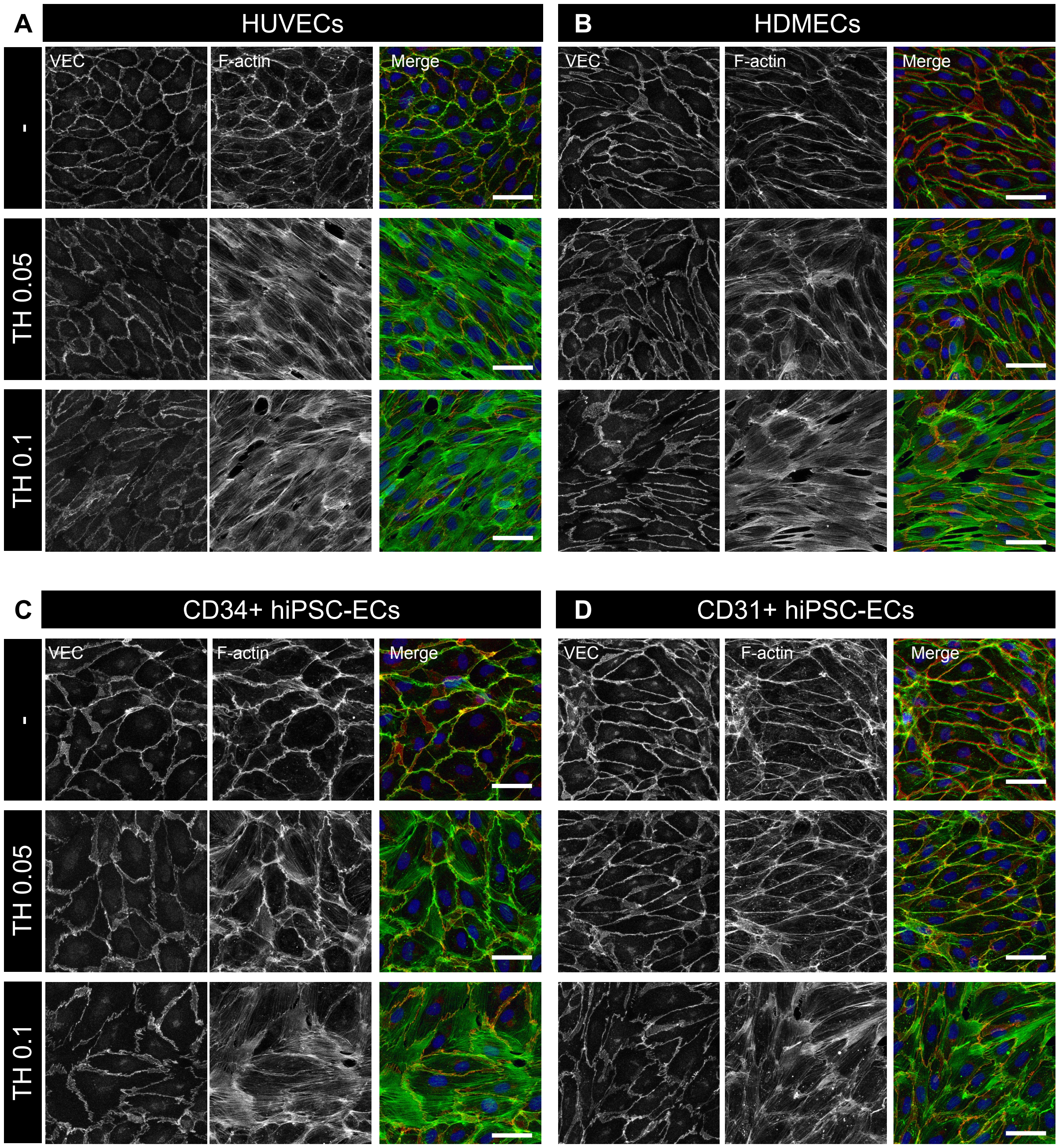

Figure S4. Comparison of junctional integrity in primary and hiPSC-ECs.
(A-D) Junctional integrity in primary cells and hiPSC-ECs was analysed using adherens junctional marker (VEC) counterstained with F-actin in HUVECs (A), HDMECs (B), CD34+ hiPSC-ECs (C) and CD31+ hiPSC-ECs (D) upon control stimulation with medium only (-) or thrombin (0.05U/ml and 0.1U/ml) for 30min. Disassembly of cell junctions and reorganisation of cortical actin and actin stress fibres formation can be observed in HUVECs and HDMECs upon thrombin (0.05U/ml and 0.1U/ml) stimulation. CD34+ hiPSC-ECs and CD31+ hiPSC-ECs shown robust response upon thrombin (0.1U/ml) stimulation. Representative pictures are shown from experiments performed three batches of CD31+ and CD34+ hiPSC-ECs, HUVECs from three batches (two donors, and two independent batches for one of the donors), HDMECs a single donor. Scale bar 50μm.

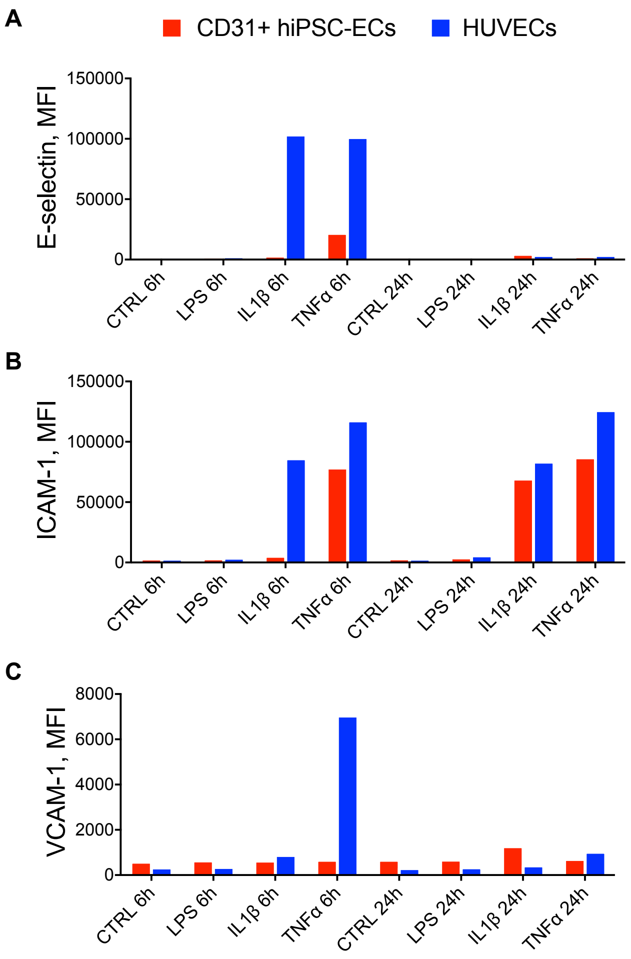

Figure S5. Related to Figure 5. Assessment of inflammatory responses in primary and hiPSC-ECs. (A-C) FACs analysis of surface expression of E-selectin (A), ICAM-1 (B) and VCAM-1 (C) after 6h and 24h post-treatment with LPS (100ng/ml), IL1β (10ng/ml) and TNFα (10ng/ml).

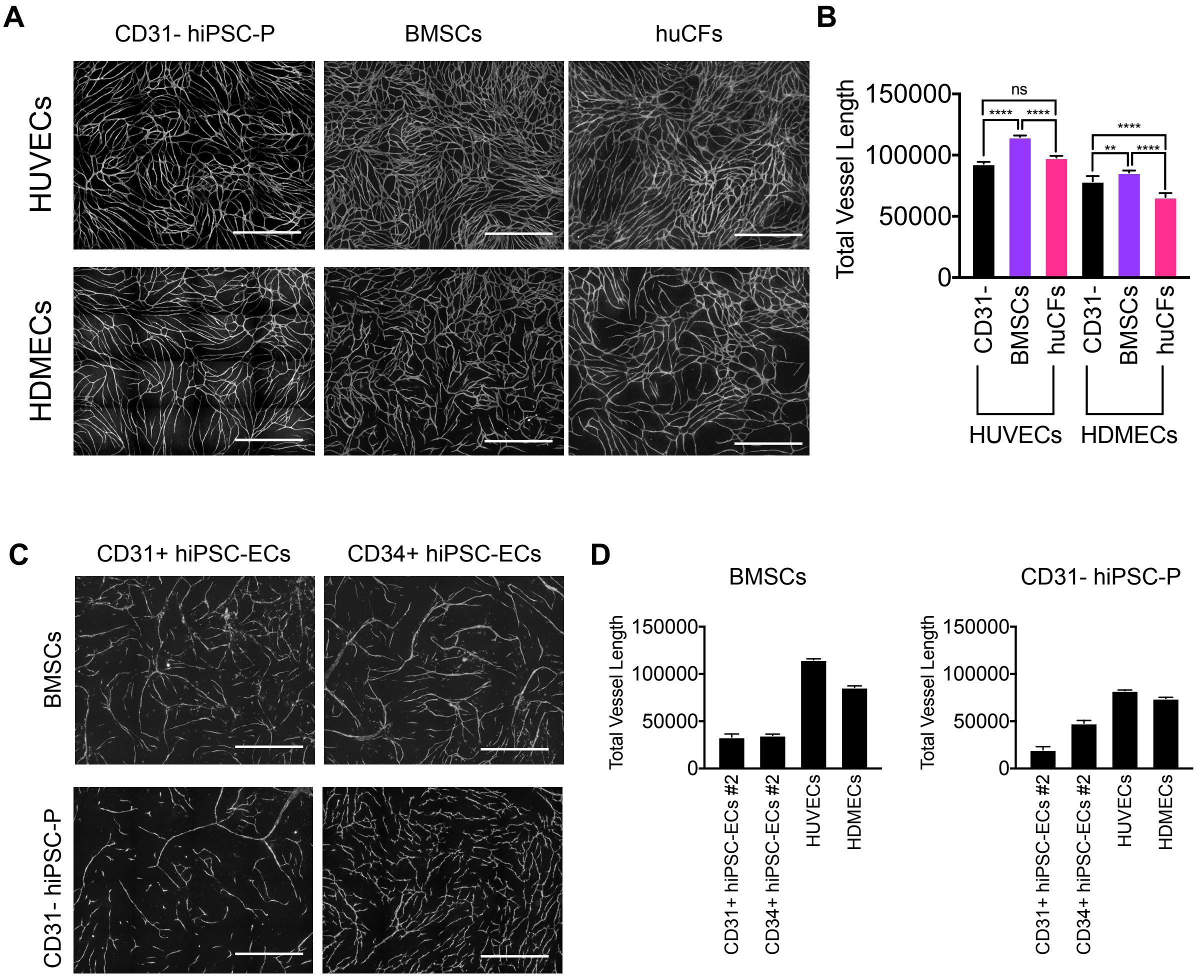

Figure S6. Related to Figure 6. Comparison the effect of different stroma cells in an *in vitro* vasculogenesis assay. (A) Representative immunofluorescent images of an *in vitro* vasculogenesis sprouting assay at day 10 of the co-culture of primary ECs (HUVECs and HDMECs) and different stroma cells (CD31- hiPSC-P, BMSCs and huCFs) used for quantification of the sprouting network. ECs are visualized with anti-CD31 (white). Automatically stitched images (10X objective, 4X4 focus planes) are shown. The images were taken with an automated imaging system with autofocus on CD31. **(B)** Quantification of EC sprouting network at day10 of the co-culture. Quantification was performed with Angiotool software. The total vessel length and total number of junctions are shown. Automatically stitched images (10X objective, 4X4 focus planes) from four to five co-cultures were used for quantification. Data are shown as ±SD. **(C)** Representative immunofluorescent images of an *in vitro* vasculogenesis sprouting assay at day 10 of the co-culture of CD31+ and CD34+ hiPSC-ECs with BMSCs and CD31- hiPSC-P used for quantification of the sprouting network. ECs are visualized with anti-CD31 (white). Automatically stitched images (10X objective, 4X4 focus planes) are shown. The images were taken with an automated imaging system with autofocus on CD31. **(D)** Quantification of EC sprouting network at day10 of the co-culture. Quantification was performed with Angiotool software. The total vessel length and total number of junctions are shown. Automatically stitched images (10X objective, 4X4 focus planes) from five co-cultures were used for quantification. Scale bar 1000μm. Data are shown as ±SD.

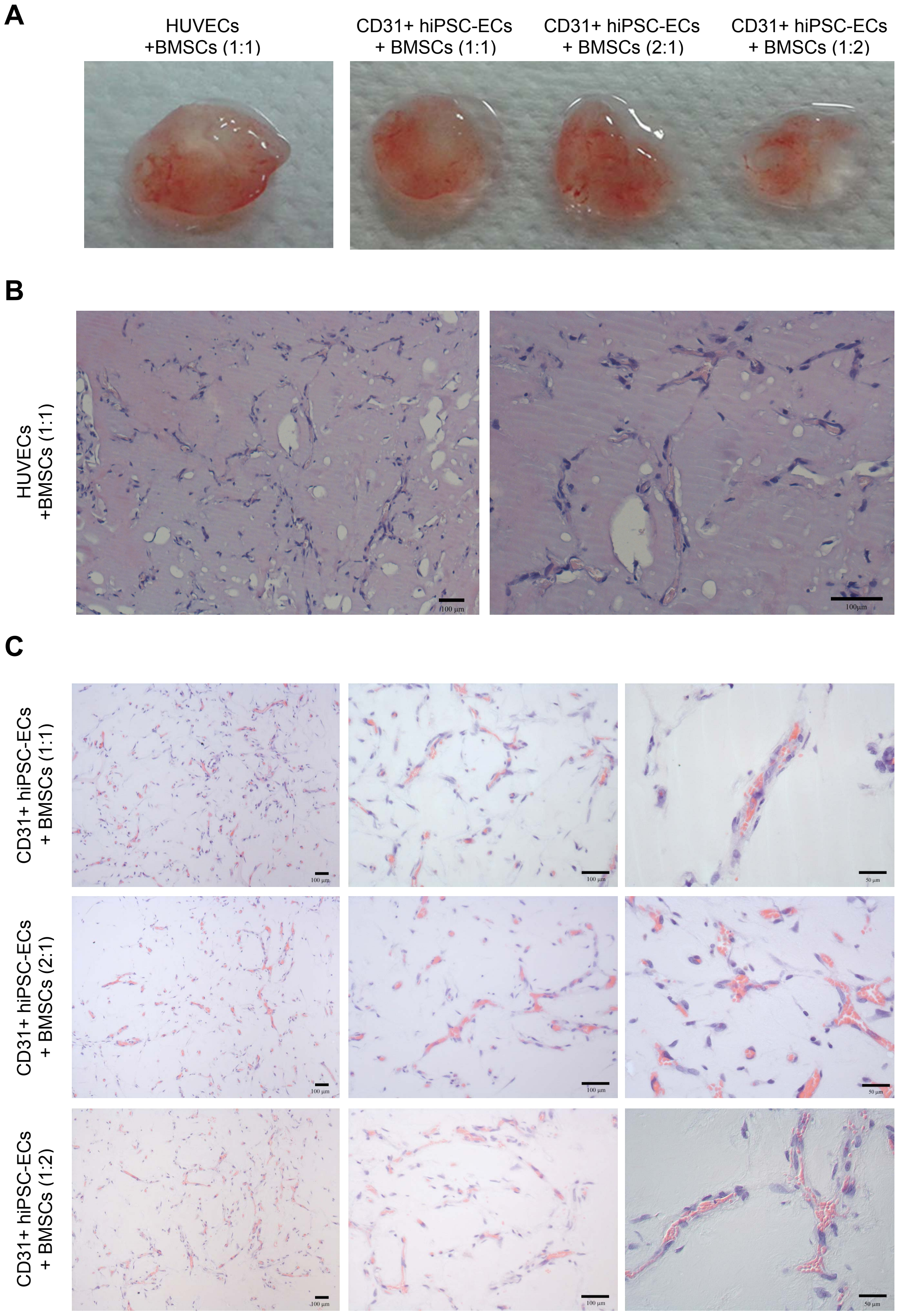

Figure S7. Related to Figure 7. Comparison of primary and hiPSC-ECs in an *in vivo* vasculogenesis assay. (A) Representative pictures of Martigel plugs 3 weeks post transplantation. **(B)** H&E images of Matrigel plugs. Representative images of Matrigel plugs with HUVECs and BMSCs (1:1). Scale bar 100μm. **(C)** Representative H&E images of Matrigel plugs with different ratios of hiPSC-ECs and BMSCs. Scale bar 100μm and 50μm.

Supplemental Tables

**Supplemental Table 1.** Sequence of primes used for qPCR			
**Gene**	**Forward sequence**	**Reverse sequence**	**Product size**
hARP	CACCATTGAAATCCTGAGTGATGT	TGACCAGCCCAAAGGAGAAG	116
VEC	GGCATCATCAAGCCCATGAA	TCATGTATCGGAGGTCGATGGT	100
VEGFR2	CCATCTCAATGTGGTCAACCTTCT	TCCTCAGGTAAGTGGACAGGTTTC	107
VEGFR3	CTGTGCCTGCGACTGTG	GGTGTCGATGACGTGTGACT	111
NOTCH1	ATAGTCTGCCACGCCTCTG	AGTGTGAAGCGGCCAATG	148
NOTCH4	GTGGTCATGGGTGTGGATT	CAGCAAGGAAGCGGAGTAG	94
JAG1	ACTGTCAGGTTGAACGGTGTC	ATCGTGCTGCCTTTCAGTTT	92
DLL4	TATGTGTGCCAGCCAGATG	ATGACAGCCCGAAAGACAG	90
SOX17	CGAGTTGAGCAAGATGCTGG	TTGTAGTTGGGGTGGTCCTG	120
NRP1	AACACCAACCCCACAGATG	AAGTTGCAGGCTTGATTCG	82
CX40	AATCAGTGCCTGGAGAATGG	CGAACCTGGATGAAACCTTC	146
EPHRINB2	GAAGTACGAGCCCCACAGA	CCCAACGCAGAAATAAACG	91
EPHB4	GAAAAGGAAGTGCCCAACA	CTGGCAAGGGAGTCACACT	99
APLNR	TTCTGCAAGCTCAGCAGCTA	GGTGCGTAACACCATGACAG	207
COUPTFII	GCTTTCCACATGGGCTACAT	CAAGTGGAGAAGCTCAAGGC	117

Supplemental Movies

Supplemental Movie 1. Related to Figure 5. Leukocyte adhesion to TNFα treated hiPSC-ECs.

Supplemental Experimental Procedures

Differentiation of hiPSCs towards ECs
hiPSCs were maintained in mTeSR-1 or mTeSR-E8 and differentiated towards ECs using previously published protocols (Orlova et al., 2014a; 2014b). For mesoderm induction (day 0-3), either combination of BMP4 (30ng/ml), ActA (25ng/ml) and CHIR (1.5μM) or CHIR (8μM) in B(P)EL were used, with the cells plated on MT or VN-coated plates respectively. The cultures were refreshed with vascular specification medium comprised of VEGF (50ng/ml) and SB431542 (10μM) in B(P)EL at day 3, day 6, and day 9. CD34+ ECs were isolated at day 6 of differentiation using EasySep™ CD34 Human Cord Blood Isolation Kit II (SCT) according to manufacturer's custom protocol (Giacomelli et al., 2017a; 2017b). CD31+ ECs were isolated at day 10 of differentiation using CD31 Dynabeads (Thermo Fisher Scientific), as previously described (Orlova et al., 2014b; 2014a). CD34+ hiPSC-ECs were plated post-isolation at the seeding density ~8,000cells/cm2 on fibronectin (FN)-coated plates, and CD31+ hiPSC-ECs were plated post-isolation at the seeding density ~12,000cells/cm2 on gelatin-coated plates (Orlova et al., 2014b). hiPSC-ECs were expanded in complete EC growth medium comprised of Human Endothelial-SFM (EC-SFM) with 1% platelet poor serum, VEGF (30ng/ml) and bFGF (20ng/ml), as described previously (Orlova et al., 2014b; 2014a). The cells were expanded for additional 3-4 days post-isolation and cryopreserved using serum-free cryopreservation medium (CryoStor™CS10)(SCT). For characterization and functional assays CD34+ and CD31+ hiPSC-ECs were thawed and cultured on FN or gelatin-coated plates respectively in complete EC growth medium.

Assessment of hiPSC-ECs functionality in an *in vitro* vasculogenesis assay
The co-culture experiments with hiPSC-ECs or primary ECs and stromal cells were performed essentially as previously described (Orlova et al., 2014b; 2014a). The following stromal cells were used in this study: CD31- hiPSC-P, derived as described (Orlova et al., 2014b; 2014a); human BMSCs and human cardiac fibroblasts were purchased from Promocell and cultured in the medium recommended by the cell supplier according to the supplier's protocol. The co-cultures were stopped at day 10 and post-fixed and stained with anti-CD31 (DAKO) and anti-SOX17 (R&D) antibodies. The co-cultures were imaged with the EVOS FL AUTO2 Imaging system (ThermoFischer Scientific) with the 10X Objective for quantifications with autofocus on CD31, and auto stitching 4X4 focus planes or 20X Objective for CD31 and SOX17 images. The co-cultures were quantified using publicly available software AngioTool (Zudaire et al., 2011).

Endothelial barrier function analysis
Endothelial barrier function analysis was performed using impedance-based cell monitoring using electric cell-substrate impedance sensing system (ECIS Zθ, Applied Biophysics). CD34+ and CD31+ hiPSC-ECs were seeded on FN-coated ECIS arrays each containing 8 wells with 10 gold electrodes per well (8W10E PET, Applied Biophysics). The cell seeding density was estimated ~50,000cells/cm^2. For barrier function and migration studies the cells were seeded for at least 24h in complete EC growth medium followed by 6h serum starvation step in EC-SFM. For the assessment of cell migration after serum starvation, the medium was changed to EC-SFM or EC-SFM supplemented with VEGF 75ng/ml, and electric wound (10 sec pulse of 5V at 60 kHz) was applied to the cells 1h after medium change. Recovery of the barrier was monitored in real time over 6-12h. Multiple frequency/time (MFT) mode was used for the real-time assessment of the barrier and monolayer confluence. To assess the disruption of the EC barrier upon administration of histamine or thrombin, the medium was first changed to EGM-2 followed by application of a new electrical wound (to replace "old" cells on the electrodes with the "new" cells), and recovery of the barrier was monitored for another 24h. The medium was changed to EBM-2 (basal medium) 1-2h prior to stimulation. Histamine or thrombin stimulation was performed at single frequency/time (SFT) mode at 4kHz by removal of 100μl of growth medium and adding first 100μl of EBM-2 (negative control) followed by ~30min recording, removal of 100μl growth medium and adding 100μl of 4X concentrated stock of histamine to final concentration 10μM followed by 1h recording and removal of 100μl growth medium and adding 100μl of 4X concentrated stock of thrombin in EBM-2 medium with the end concentration (0.05 and 0.1U/ml).

Assessment of junctional integrity
Analysis of junctional integrity was performed as previous described (Orlova et al., 2006) with some modifications. Briefly, ECs were seeded on FN-coated 96-well black imaging plates (Corning) at the seeding density ~10,000cells/well in EGM-2 (primary ECs) or complete EC-SFM (hiPSC-ECs) medium. 48h post-seeding ECs were serum-starved in 100μl EBM-2 medium followed by thrombin stimulation by adding 100μl of 2X concentrated stock of thrombin in EBM-2 medium with the end concentration

(0.05 and 0.1U/ml). Cells were fixed with 4% paraformaldehyde (PFA, Sigma), permeabilized with the 0.1%TX-100 and stained with anti-ZO1 (ThermoFisher) or VEC (CellSignaling) and counterstained with A488 conjugated Phalloidin (ThermoFisher). High magnification images were acquired with the WLL1 confocal microscope (Leica), using 40x DRY objective using 0.75 Zoom factor.

Stimulation with pro-inflammatory cytokines
ECs were stimulated in complete EC growth medium with pro-inflmmatory cytokines (TNFα 10ng/ml, IL1β 10ng/ml) or LPS (100ng/ml) in complete EC growth medium. FACs analysis of the expression of E-selectin, ICAM-1 and VCAM-1 was performed at 6, 12 and 24h post-stimulation.

Flow adhesion assay for leukocyte-endothelial cell interaction
Vena8 Endothelia+ chips (Cellix) were coated with FN (50μg/ml) overnight (ON) at 4°C in a humidified chamber by injecting ~10μl into the microfluidic channel. ECs were stimulated in complete EC growth medium with TNFα (10ng/ml) for ~12h (ON). Next day ECs were detached, counted and re-suspended in EGM-2 medium (HUVECs) or complete EC-SFM (hiPSC-ECs) at ~1.5E6 cells/100μl. ECs were seeded into a microfluidic chip by injecting ~6μl of the cell suspension into the microfluidic channel. Microfluidic chips were incubated at 37°C for ~15min in a humidified chamber in order to facilitate cell attachment, and additional 40μl of medium was added from the both sides of the channel. The cells were incubated for 1h at 37°C, after 1h ~50-80μl of the medium was added from both sides of the channel and the chips were kept at 37°C prior to the assay. The assay was performed within ~2h of cell seeding. Human leukocytes (THP1) were washed once with PBS and re-suspend in 1ml of PBS with DiOC6 (1:5000)(Sigma). THP1 cells were incubated in the dark at RT for 10min at RT, washed with PBS and re-suspend in complete RPMI medium at the end concentration 2.5E6 cells/ml. THP1 cells for flow adhesion experiments were perfused for 5 minutes at 0.5 dyne/cm2, followed by a 5 min wash with RPMI medium. The number of adherent fluorescently labelled THP1 cells on ECs was quantified using CellProfiler (Carpenter et al., 2006).

Assessment of hiPSC-ECs functionality in an *in vitro* vasculogenesis assay
The co-culture experiments with hiPSC-ECs or primary ECs and stromal cells were performed essentially as previously described (Orlova et al., 2014b; 2014a). Details are provided in supplemental experimental procedures.

Transplantation of hiPSC-ECs and BMSCs
The Matrigel plug assay using hiPSC-ECs and BMSCs was performed as previously described (Sacchetti et al., 2016). Experiments were carried out in compliance with relevant Italian laws and Institutional guidelines and all procedures were IACUC approved. hiPSC-ECs and BMSCs were suspended in 1 ml GF-reduced Matrigel (BD Biosciences Labware) at different ratios: 1 million hiPSC-ECs and one million BMSCs (1:1), 2 million hiPSC-ECs and 1 million BMSCs (2:1), 1 million hiPSC-ECs and 2 million BMSCs (1:2). In control samples, 1 million HUVECs were mixed with 1 million BMSCs. Cell suspensions (~ 0.7 ml) were injected subcutaneously in the back of SCID/beige mice (CB17.Cg-Prkdcscid Lyst bg-J/Crl; Charles River). Three weeks after transplantation, the plugs were harvested, fixed in 4% neutral buffered formaldehyde for 24hr at 4°C and routinely embedded in paraffin. Five-micron-thick sections were cut from paraffin blocks for hematyoxilin and eosin (H&E) staining and immunohistochemistry. For human CD31 immunolocalization, deparaffinized sections were incubated with mouse anti-Human CD31 antiserum (Endothelial Cell Clone JC70A; M0823 Dako) diluted 1:30 in phosphate buffered saline (PBS) for 2h at RT, washed with PBS and then exposed for 30 min' at RT to Alexafluor goat anti Mouse IgG1 488 (A-21121; ThermoFisher Scientific) diluted 1:200 in PBS. For DNA counterstaining, sections were incubated for 15 min at RT with Topro-3 (T3605 ThermoFisher Scientific) diluted1:1000 in PBS.

Transplantation of hiPSC-ECs and CD31- hiPSC-P
Matrigel plug assay with hiPSC-ECs and CD31- hiPSC-P was performed similar to BMSCs transplantation experiments with minor modifications. Animal experiments were approved by the Leiden University Medical Centre animal experimental committee and the Commission Biotechnology in Animals of the Dutch Ministry of Agriculture. Two million hiPSC-ECs and one million CD31- hiPSC-P were mixed in 600 µl of GF-reduced Matrigel (BD Biosciences Labware), supplemented with bFGF (1ug/ml), VEGF (200ng/ml) and Heparin (2.5U) and injected subcutaneously in the back region of 8-10 weeks old male NSG mice (NOD.Cg-Prkdc^{scid} Il2rg^{tm1Wjl}/SzJ, Charles River). Three mice were used per group. After cell injection, each mouse received subcutaneous injections of 100ng/μl bFGF every 48

hours. Three weeks after transplantation, the plugs were harvested, and immersed in 4% formaldehyde in Phosphate-Buffered solution at RT for 4h, followed 15% Sucrose-PBS solution for a minimum of 2h and subsequently to 30% Sucrose-PBS solution O/N at 4°C. Plugs were embedded in Tissue-Tek® OCT compound (Sakura® Finetek) for further analysis. Eight-micron-thick frozen sections were processed for H&E. For immunofluorescence staining sections were fixed with PBS/2% formaldehyde for 10 minutes at RT, permeabilized for 8 minutes with PBS/0.1% Triton-X-100 and incubated with the primary antibody were at 4°C O/N, followed by the secondary antibody for 1 hour at RT (for antibodies see Table S1). DNA counterstaining was performed with DAPI (1:1000, company). To quantify labelling for mouse and human specific fluorescence, labelled sections were digitalized using the Pannoramic Viewer software (3DHistech). For each plug 3 to 4 sections at 80-100 um distance were analyzed. In total 11 pictures per plug (20X magnification) were analyzed. The vascular density of human CD31 positive cells and CD31 mouse and human positive cells was measured by determining the area percentage above threshold on a large representative set of images. The threshold was determined interactively and was kept constant for the complete set. The analysis was performed using an in-house image analysis package, called Stacks.

Supplemental References

Carpenter, A.E., Jones, T.R., Lamprecht, M.R., Clarke, C., Kang, I.H., Friman, O., Guertin, D.A., Chang, J.H., Lindquist, R.A., Moffat, J., Golland, P., Sabatini, D.M., 2006. CellProfiler: image analysis software for identifying and quantifying cell phenotypes. Genome Biology 7, R100. doi:10.1186/gb-2006-7-10-r100

Giacomelli, E., Bellin, M., Orlova, V.V., Mummery, C.L., 2017a. Co-Differentiation of Human Pluripotent Stem Cells-Derived Cardiomyocytes and Endothelial Cells from Cardiac Mesoderm Provides a Three-Dimensional Model of Cardiac Microtissue. Curr Protoc Hum Genet 95, 21.9.1–21.9.22. doi:10.1002/cphg.46

Giacomelli, E., Bellin, M., Sala, L., van Meer, B.J., Tertoolen, L.G.J., Orlova, V.V., Mummery, C.L., 2017b. Three-dimensional cardiac microtissues composed of cardiomyocytes and endothelial cells co-differentiated from human pluripotent stem cells. Development dev.143438–47. doi:10.1242/dev.143438

Orlova, V.V., Drabsch, Y., Freund, C., Petrus-Reurer, S., van den Hil, F.E., Muenthaisong, S., Dijke, P.T., Mummery, C.L., 2014a. Functionality of endothelial cells and pericytes from human pluripotent stem cells demonstrated in cultured vascular plexus and zebrafish xenografts. Arteriosclerosis, Thrombosis, and Vascular Biology 34, 177–186. doi:10.1161/ATVBAHA.113.302598

Orlova, V.V., Economopoulou, M., Lupu, F., Santoso, S., Chavakis, T., 2006. Junctional adhesion molecule-C regulates vascular endothelial permeability by modulating VE-cadherin-mediated cell-cell contacts. Journal of Experimental Medicine 203, 2703–2714. doi:10.1084/jem.20051730

Orlova, V.V., van den Hil, F.E., Petrus-Reurer, S., Drabsch, Y., Dijke, ten, P., Mummery, C.L., 2014b. Generation, expansion and functional analysis of endothelial cells and pericytes derived from human pluripotent stem cells. Nature Protocols 9, 1514–1531. doi:10.1038/nprot.2014.102

Sacchetti, B., Funari, A., Remoli, C., Giannicola, G., Kogler, G., Liedtke, S., Cossu, G., Serafini, M., Sampaolesi, M., Tagliafico, E., Tenedini, E., Saggio, I., Robey, P.G., Riminucci, M., Bianco, P., 2016. No Identical "Mesenchymal Stem Cells" at Different Times and Sites: Human Committed Progenitors of Distinct Origin and Differentiation Potential Are Incorporated as Adventitial Cells in Microvessels. STEMCR 6, 897–913. doi:10.1016/j.stemcr.2016.05.011

Zudaire, E., Gambardella, L., Kurcz, C., Vermeren, S., 2011. A Computational Tool for Quantitative Analysis of Vascular Networks. PLoS ONE 6, e27385–12. doi:10.1371/journal.pone.0027385
