# Supplementary material for: Inflammatory Responses and Barrier Function of Endothelial Cells Derived from Human Induced Pluripotent Stem Cells
Source: Stem Cell Reports. 2018 Apr 12;10(5):1642–56. doi: 10.1016/j.stemcr.2018.03.012 (PMC5995303; doi:10.1016/j.stemcr.2018.03.012)
Supplement: Document S2. Article plus Supplemental Information [file mmc3.pdf]

Stem Cell Reports
Article

OPEN ACCESS

Inflammatory Responses and Barrier Function of Endothelial Cells Derived from Human Induced Pluripotent Stem Cells

Oleh V. Halaidych,[1] Christian Freund,[1] Francijna van den Hil,[1] Daniela C.F. Salvatori,[2] Mara Riminucci,[3] Christine L. Mummery,[1] and Valeria V. Orlova[1,*]

[1]Department of Anatomy and Embryology, Leiden University Medical Center, Einthovenweg 20, 2333ZC Leiden, the Netherlands
[2]Central Laboratory Animal Facility, Einthovenweg 20, 2333ZC Leiden, the Netherlands
[3]Department of Molecular Medicine, Sapienza University of Rome, Rome, Italy
*Correspondence: v.orlova@lumc.nl
https://doi.org/10.1016/j.stemcr.2018.03.012

SUMMARY

Several studies have reported endothelial cell (EC) derivation from human induced pluripotent stem cells (hiPSCs). However, few have explored their functional properties in depth with respect to line-to-line and batch-to-batch variability and how they relate to primary ECs. We therefore carried out accurate characterization of hiPSC-derived ECs (hiPSC-ECs) from multiple (non-integrating) hiPSC lines and compared them with primary ECs in various functional assays, which included barrier function using real-time impedance spectroscopy with an integrated assay of electric wound healing, endothelia-leukocyte interaction under physiological flow to mimic inflammation and angiogenic responses in *in vitro* and *in vivo* assays. Overall, we found many similarities but also some important differences between hiPSC-derived and primary ECs. Assessment of vasculogenic responses *in vivo* showed little difference between primary ECs and hiPSC-ECs with regard to functional blood vessel formation, which may be important in future regenerative medicine applications requiring vascularization.

INTRODUCTION

Human induced pluripotent stem cells (hiPSCs) can be derived by reprogramming somatic cells from any individual. The ability to derive different cell types of the body and scale production has generated interest in their use in drug discovery, disease modeling, and regenerative medicine (Passier et al., 2016; Samuel et al., 2015; Shi et al., 2016). DNA-free reprogramming methods, where the reprogramming vectors are not integrated into the genome, are now considered to show the lowest risk of targeting important genes unintentionally. Sendai virus (SeV)-based reprogramming in particular has been widely used to generate hiPSCs from skin fibroblasts (FiPSCs), nasal epithelial cells, peripheral blood mononuclear cells (MNCs), and cells in urine (UiPSCs) (Chen et al., 2013; Fusaki et al., 2009; Hildebrand et al., 2016; Ono et al., 2012). Cells in human urine are proving of increasing interest since they can be collected non-invasively and thus from children or others preferring not to donate blood or a skin biopsy. We and others have generated endothelial cells (ECs) from hiPSC lines from these different somatic cell types, including UiPSCs (Cai et al., 2015; Orlova et al., 2014a; Patsch et al., 2015; Rufaihah et al., 2013; Zhang et al., 2017). However, to date there have been few direct comparisons with primary human ECs in robust assays for assessing functionality, and hiPSC-derived ECs (hiPSC-ECs) have not been compared for line-to-line and batch-to-batch variability. This has limited their utility in disease modeling and drug discovery, particularly where isogenic controls for patient lines are not available since it may be difficult to distinguish line-to-line "noise" from true, disease-related phenotypes. Furthermore, widely available human umbilical vein ECs (HUVECs) are often used in preference to hiPSC-ECs in bioassays since they are perceived as more robust, but functional comparisons are rarely made (Iwata et al., 2017). Exceptionally, we showed that the ability of HUVECs to integrate into the developing vasculature (in zebrafish) is inferior to that of hiPSC-ECs (Orlova et al., 2014a). Here, we have undertaken direct side-by-side comparison of hiPSC-ECs with primary ECs, such as human dermal blood ECs (HDMECs) and HUVECs, in several widely used functional *in vitro* and *in vivo* assays. Two independent "bead-based" methods were used for hiPSC-EC isolation: CD34 + cells on day 6 of differentiation and CD31 + cells on day 10. Multiple batches of ECs were compared among a range of isogenic and non-isogenic hiPSC lines.

Barrier function was chosen as one assay that would likely be comparable across a wide set of isogenic and non-isogenic hiPSC-ECs in confluent cultures if the cells were derived in the same way. Two principal mechanisms contribute to the regulation of the EC barrier: transcellular and paracellular permeability. Paracellular permeability, or opening of inter-endothelial junctions, is linked to many pathological processes, including acute vascular leak syndrome or sepsis, acute respiratory distress syndrome, anaphylactic shock, and tumor angiogenesis. Impedance-based techniques, such as electric cell-substrate impedance sensing (ECIS), provide accurate and sensitive methods to measure endothelial barrier function, including rapid

This is an open access article under the CC BY license (http://creativecommons.org/licenses/by/4.0/).

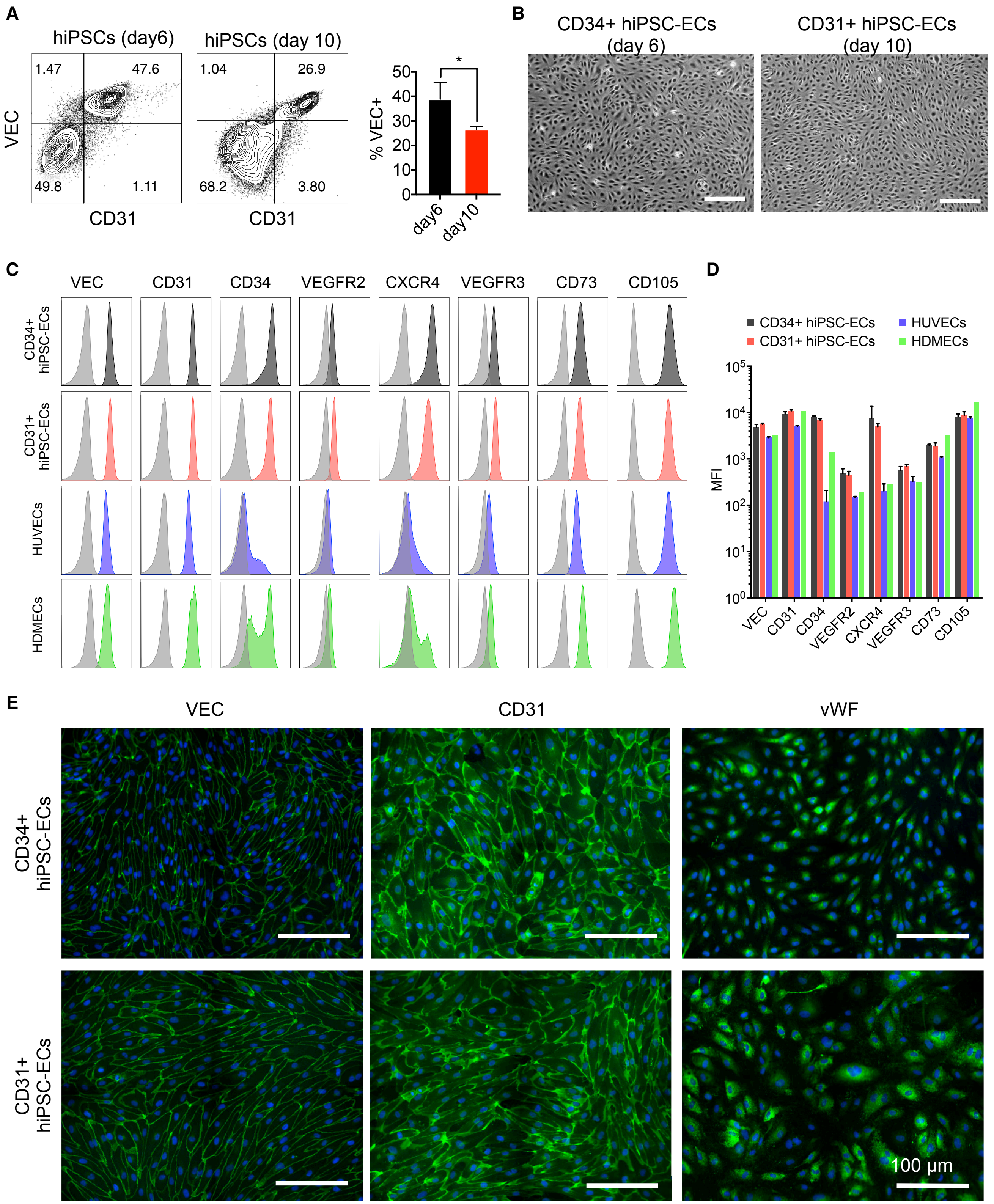

(legend on next page)

changes upon stimulation with barrier-disrupting agents, such as thrombin or histamine or known barrier-elevating agents, such as cyclic AMP (Stolwijk et al., 2014). Despite many reports on the generation of hPSC-ECs, only a few studies have evaluated barrier function using impedance sensing (Adams et al., 2013; Patsch et al., 2015). Here, we compared hiPSC-ECs with primary ECs in barrier function assays that included examining the disruptive effects of histamine and thrombin. These factors are known to cause transient increases in endothelial permeability, disassembly of inter-endothelial cell-cell junctions, and decrease in barrier function in primary ECs.

Secondly, inflammatory responses were examined. Heterogeneity in inflammatory responses has been reported among different vascular beds, and types of ECs (Aird, 2012). ECs play essential roles in regulating inflammation by limiting leukocyte extravasation at the site of injury/inflammation, as in the case of non-inflamed/healthy endothelium, or facilitating extravasation upon local tissue injury or inflammation. The leukocyte recruitment cascade and molecular players that regulate these processes are well characterized and include pro-adhesive receptors, such as E-selectin, intercellular adhesion molecule-1 (ICAM-1), and vascular cell adhesion molecule-1 (VCAM-1). These receptors are upregulated on the EC surface and participate in capturing and "rolling" leukocytes on the vessel wall, to mediate firm adhesion (Hajishengallis and Chavakis, 2013; Nourshargh and Alon, 2014). The transmigration of leukocytes is further mediated via interplay with homotypic cell adhesion receptors, such as vascular endothelial cadherin (Ve-cadherin), junctional adhesion molecules (JAMs), EC-selective adhesion molecule (ESAM), CD99, and others (Nourshargh and Alon, 2014) that are expressed between endothelial cell-cell junctions. Chronic inflammation contributes to many different pathological conditions, such as cardiovascular and neurological and neurodegenerative disorders (Passier et al., 2016). Uncontrolled or systemic inflammation results in severe pathological conditions such as sepsis, or adverse drug responses. Thus, careful assessment of inflammatory responses in hiPSC-ECs is needed before decisions can be made on their utility in future assays on, for example, the effects of genetic background on inflammatory responses in patient-specific hiPSC-derived tissues or regenerative medicine.

We carried out extensive assessment of hiPSC-ECs from multiple hiPSC lines and batches in all of the assays described above (barrier function, transient disruption of barrier, expression of inflammatory adhesive receptors, and leukocyte adhesion under flow) and compared them with primary ECs. Finally, angiogenic/vasculogenic responses and the ability to form functional blood vessels were compared *in vitro* and *in vivo*.

RESULTS

Differentiation of hiPSCs toward ECs

hiPSC lines were generated using SeV (Nishimura et al., 2011), (Zhang et al., 2014). For differentiation toward ECs, we used a protocol based on defined reagents without serum, as previously described (Orlova et al., 2014a, 2014b). We examined the percentages of Ve-cadherin+ (VEC+) cells on day 6 and day 10 of differentiation and found this was significantly higher on day 6 compared with day 10 (Figure 1A), in agreement with our previous findings (Giacomelli et al., 2017; Orlova et al., 2014a). In order to isolate ECs, CD34 and CD31 magnetic-bead-based purification was used on day 6 and day 10 of differentiation, respectively, as described previously (Giacomelli et al., 2017; Orlova et al., 2014a, 2014b). ECs isolated either on day 6 or day 10 displayed typical EC-like morphology (Figure 1B). Fluorescence-activated cell sorting (FACS) analysis of CD34+ and CD31+ hiPSC-ECs revealed their comparable expression of known EC surface markers, such as VEC, CD31, CD34, VEGFR2, CXCR4, VEGFR3, CD73, and CD105 (Figures 1C and 1D). Expression of VEC, CD31, CD73, and CD105 by CD34+ and CD31+ hiPSC-ECs was also similar to that in primary HUVECs and HDMECs, while expression of CD34, CXCR4, VEGFR2, and VEGFR3 was higher. Gene expression

Figure 1. Differentiation of hiPSCs toward ECs

(A) Representative FACS plots and quantification of the percentage of VEC+ cells at day 6 and day 10 differentiation of UiPSCs. Average %VEC+ from three independent biological replicates are shown, error bars represent ±SD. *$p < 0.05$.

(B) Phase-contrast images of CD34+ and CD31+ hiPSC-ECs 3 days post isolation. Scale bar represents 300 μm.

(C) FACS analysis of surface marker expression on isolated CD34+ and CD31+ hiPSC-ECs at passage 2 (P2) and primary ECs (HUVECs and HDMECs at P4–P5). Black and color filled histograms are staining with the antibody of interest; light gray histograms are relevant isotype control.

(D) Quantification of surface marker expression on isolated CD34+ and CD31+ hiPSC-ECs at passage 2 (P2). Median fluorescence intensity values are shown for three batches of CD31+ and CD34+ hiPSC-ECs, HUVECs from three batches (two donors, and two independent batches for one of the donors) and HDMECs from a single donor. Error bars represent ±SD.

(E) Immunofluorescent analysis of EC markers VEC, CD31, and vWF on isolated CD34+ and CD31+ hiPSC-ECs (P2). Scale bar represents 100 μm.

profiling revealed a mixed arterial- and embryonic-like identity in hiPSC-ECs with prominent expression of both arterial markers, such as *VEGFR2* (*KDR*) and *SOX17*, and venous markers, such as *COUPTFII* (*NR2F2*) and *APLNR*. However, expression of other well-established arterial markers, such *NOTCH1*, *NOTCH4*, *JAG1*, *NRP1*, *CX40* (*GJA5*), and *EPHRINB2* (*EFNB2*), was lower than in human umbilical artery ECs (HUAECs) (Figure S1A). Immunofluorescent staining revealed inter-junctional localization of VEC, CD31, and ZO1, and intracellular von Willebrand factor (vWF) (Figures 1E and S1B), although overall vWF levels were lower compared with primary ECs (Figure S1B).

Comparative Assessment of Barrier Function and Real-Time Migration of Primary and hiPSC-ECs

Barrier function and EC migration were assessed by real-time impedance spectroscopy with an integrated assay of electric wound healing, shown schematically in Figure 2A. We first compared barrier function of ECs derived from several independent hiPSC lines. HDMECs from one donor, HUVECs from two independent donors, and two independent batches for one of the donors were used. Primary cells had comparable population doubling times based on data from the cell provider thus avoiding possible differences in growth rate affecting function. Importantly, we found that barrier function of SeV UiPSC and FiPSC-derived CD31 + ECs was very similar (Figures 2B and 2C). However, barrier function of CD34+ hiPSC-ECs isolated on day 6, compared with ECs isolated at day 10, was significantly lower compared with CD31+ hiPSC-ECs derived from two independent (isogenic) clones of one line, as well as another independent FiPSC line (Figures 2B and 2C). In addition, we further investigated barrier function of different batches of CD31+ and CD34+ hiPSC-ECs. We found that independent batches of CD31+ hiPSC-ECs isolated from three SeV hiPSC lines were comparable, with no significant variation among the batches (Figures S2A–S2C). On the other hand, CD34+ hiPSC-ECs (day 6) had higher batch-to-batch variability (Figure S2D). Very little variation across primary ECs was observed (Figure S2E). Thus, ECIS-based assessment of barrier function of hiPSC-ECs is a useful and reproducible quality control assay, particularly in assessing ECs derived from independent hiPSC lines, and independent batches of the same line. Furthermore, CD31+ hiPSC-ECs that are isolated on day 10 are similar, independent of line, genetic background, or batch, and thus might be the most robust readout of disease phenotype in patient hiPSC-ECs or in drug screening applications. When compared with primary ECs, such as HDMECs and HUVECs, CD31+ hiPSC-ECs exhibited either similar, as in the case of FiPSC-ECs versus HDMECs, or higher barrier when cultured in EGM-2 medium (Figure S2F). This is important, since, in contrast to primary ECs with a limited lifespan, hiPSC-ECs can be derived from any individual in unlimited numbers.

In addition, CD31+ and CD34+ hiPSC-ECs exhibited high sensitivity to VEGF (Figure 2D). Interestingly, although not observed in primary ECs, hiPSC-ECs cultured in basal serum- and growth factor-free medium exhibited increased barrier characteristics compared with "complete" growth medium containing serum (Figures 2D and 2E). Supplementation with VEGF (75 ng/mL) significantly decreased the endothelial barrier, and this was comparable with the complete growth culture medium condition. Migration rates in the real-time migration assay were lower in VEGF supplemented medium, compared with complete growth medium (Figures 2F and 2G). No significant difference was found in migration rates of CD31+ and CD34+ hiPSC-ECs in complete growth medium and VEGF supplemented medium (Figure 2G). Thus, assessment of both barrier function and migration are useful for validating hiPSC-EC functionality, including quality control of independent EC batches, media formulations, and protocols. Of clinical relevance, the assays could be used to screen for compounds that alleviate or aggravate VEGF sensitivity, an important mechanism underlying disease pathology. Somatic cell source and reprogramming methods tested here did not affect these functional characteristics.

Comparison of Barrier Disruption in Primary and hiPSC-ECs

Barrier disruption was examined as shown schematically in Figure 3A. For these experiments, hiPSC-ECs first formed confluent monolayers in complete growth medium, the medium was replaced by EGM-2 for at least 12 hr (which is compatible with the wound healing assay), and then they were serum starved in EBM-2 medium for an additional 2–3 hr, since hiPSC-ECs exhibited very poor responses to known permeability factors in complete growth medium (data not shown). EGM-2 medium was chosen as it is widely used for primary ECs. Surprisingly, we found that neither CD31 + or CD34+ hiPSC-ECs were responsive to histamine (Figures 3B and 3C). HDMECs, on the other hand, exhibited a very pronounced and rapid drop in barrier resistance as early as 1 min post stimulation. Less prominent decreases were also observed in HUVECs, but this was not significant compared with stimulation with control medium (compound free) (Figures 3B, 3C, and S3A). Stimulation of hiPSC-ECs with thrombin decreased the endothelial barrier, although only at higher concentrations (0.1 U/mL) (Figures 3B, 3C, S3B, and S3C). Comparison of CD31+ and CD34+ hiPSC-ECs revealed similar barrier disruption in response to thrombin. Despite the relatively low dosage of thrombin, hiPSC-ECs failed to recover the barrier, in contrast to

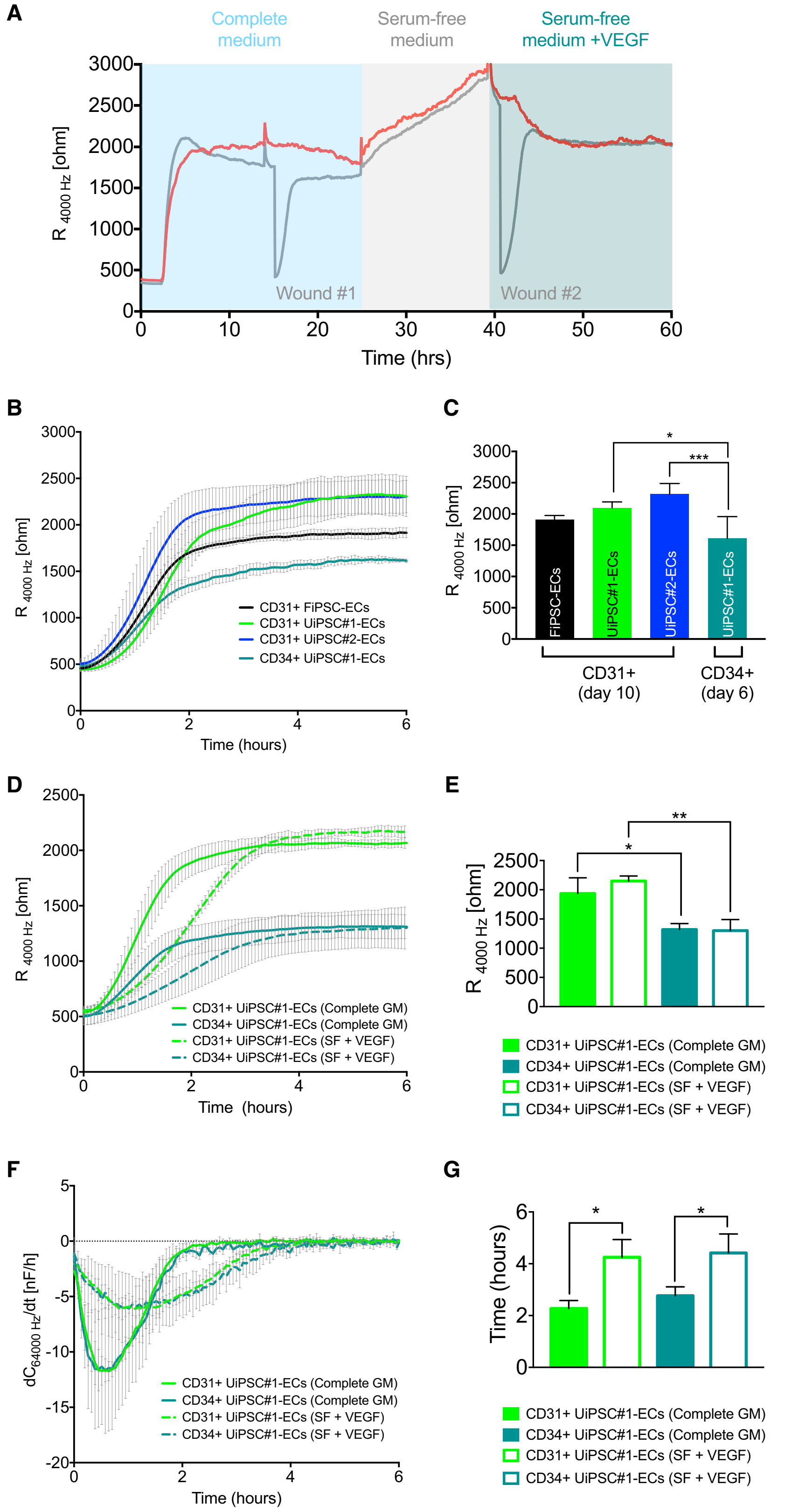

Figure 2. Comparative Assessment of Barrier Function and Real-Time Migration of Primary and hiPSC-ECs

(A) Schematic illustration of ECIS barrier function assessment and real-time migration of hiPSC-ECs.

(B) Representative absolute resistance of the EC monolayer in complete EC growth medium is shown. Error bars are shown as ±SD of three to four independent wells from representative biological experiments.

(C) Quantification of absolute resistance values at 4,000 Hz in complete EC growth medium. Values are presented as average means from a minimum of three independent biological experiments. Error bars are shown as ±SD of three independent biological experiments. $^{*}p < 0.01$, $^{***}p < 0.001$.

(D) Mean absolute resistance from of the EC monolayer in complete EC growth medium or serum-free medium supplemented with VEGF (75 ng/mL) is shown. Error bars are shown as ±SD of average values from three independent biological experiments.

(E) Quantification of absolute resistance at 4,000 Hz of the EC monolayer in complete EC growth medium or serum-free medium supplemented with VEGF (75 ng/mL). Error bars are shown as ±SD of three independent biological experiments. $^{*}p < 0.05$, $^{**}p < 0.001$.

(F) Mean speed of migration (dC/dt) determined as a change in capacitance at 64,000 Hz over the time after electric wound healing in complete EC growth medium or serum-free medium supplemented with VEGF (75 ng/mL). Error bars are shown as ±SD of average values from three independent biological experiments.

(G) Quantification of migration rates determined as a time upon closing the wound (dC/dt>(−0.1 nF/hr)) of hiPSC-ECs in real-time wound healing assay in EC monolayer in complete EC growth medium or serum-free medium supplemented with VEGF (75 ng/mL). Error bars are shown as ±SD of three independent biological experiments. $^{*}p < 0.05$.

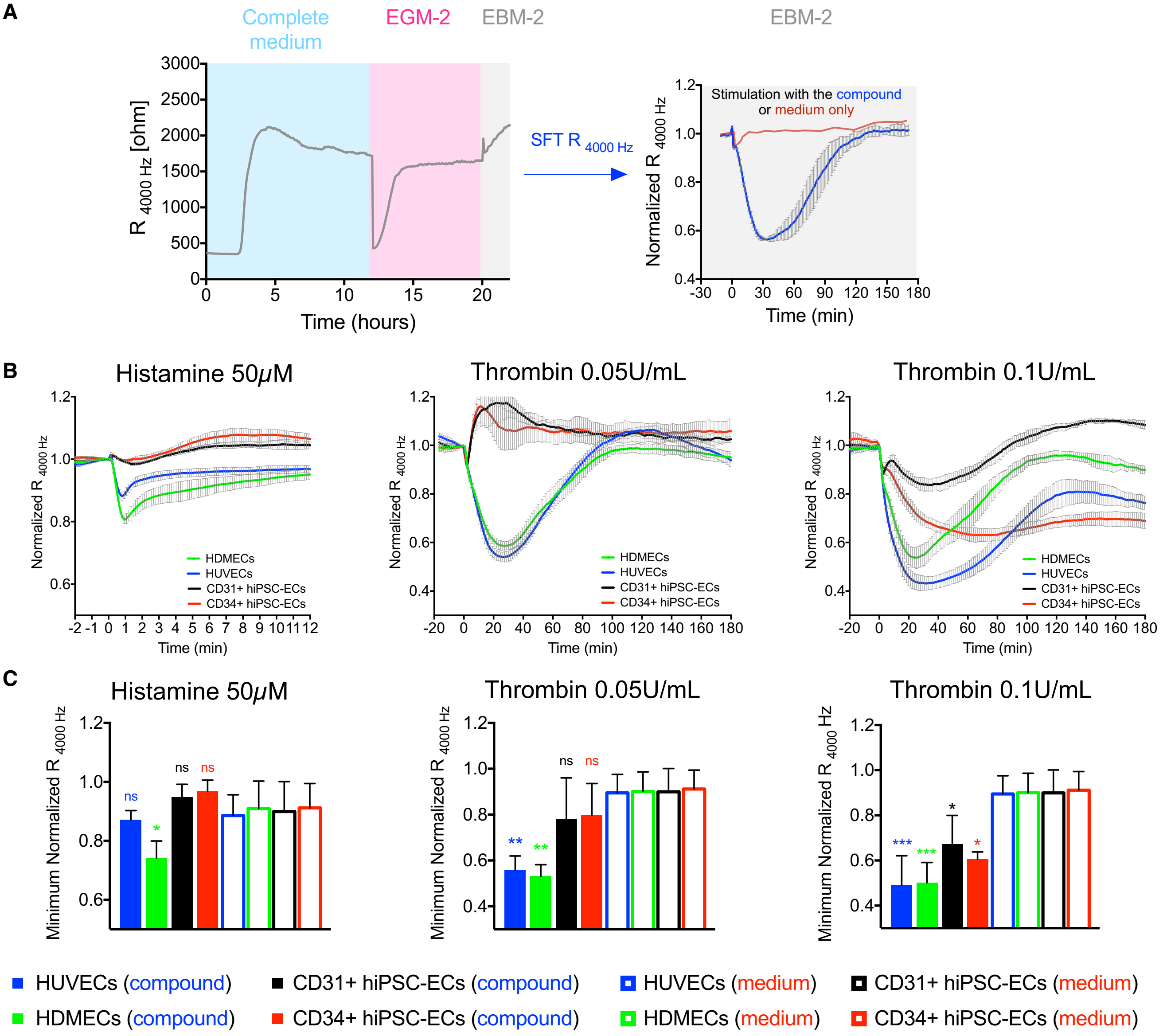

Figure 3. Comparison of Barrier Disruption in Primary and hiPSC-ECs

(A) Schematic illustration of workflow for ECIS barrier disruption assessment.

(B) Changes in normalized resistance of the EC monolayer upon stimulation with histamine (50 μM) and thrombin (0.05 U/mL and 0.1 U/mL). Stimulation time point is set as t = 0. Normalized resistance is shown as a representative plot of one representative independent experiment. Error bars are shown as ±SD of three to four independent wells.

(C) Quantification of minimal normalized resistance upon stimulation with histamine (50 μM) and thrombin (0.05 U/mL and 0.1 U/mL). Control stimulation with equal volume of medium without the compound is shown in Figure S3. Compound-mediated (filled bars) reduction in barrier function is compared with alteration of barrier upon control stimulation (empty bars). Error bars are shown as ±SD from three (n = 3) independent biological experiments. *$p < 0.05$, **$p < 0.001$, ***$p < 0.0001$.

primary ECs. In summary, we found that hiPSC-ECs responded to higher concentration of thrombin (0.1 U/mL) and were not responsive to histamine at the concentrations that disrupt the barrier in HDMECs (50 μM), or even higher (up to 200 μM, data not shown).

Comparison of Junctional Integrity in Primary and hiPSC-ECs

EC barrier function and paracellular permeability are dependent on interaction between proteins that form cell-cell junctions, mainly tight junctions (TJs) and

adherens junctions (AJs) (Giannotta et al., 2013; Komarova et al., 2017). We therefore examined organization of TJs and AJs in serum-starved hiPSC-ECs and primary ECs before and after 30 min stimulation with thrombin (0.05 U/mL and 0.1 U/mL). This time point was chosen as it coincided with the maximum decrease in barrier function evident in impedance measurements. ECs were stained with zonula occluden-1 (ZO1) and VEC to visualize TJs and AJs, respectively, and counterstained with F-actin to reveal cortical actin and formation of actin stress fibers upon junction disassembly. Primary ECs showed robust responses to thrombin (0.05 and 0.1 U/mL) associated with loss of cortical actin and formation of actin stress fibers with opening of the cell junctions (Figures 4A, 4B, S4A, and S4B), as expected from impedance measurements. Notably, hiPSC-ECs responded only to higher concentrations of thrombin (0.1 U/mL) (Figures 4C, 4D, S4C, and S4D). Furthermore, when compared in a quiescent (serum-starved) state, CD31+ hiPSC-ECs showed highly organized TJs and AJs that were similar to those in HDMECs, while CD34+ hiPSC-ECs had less organized TJs and AJs with morphology more similar to that observed in HUVECs.

Comparison of Inflammatory Response in Primary and hiPSC-ECs

hiPSC-ECs were first assayed for responses to pro-inflammatory agents, such as tumor necrosis factor alpha (TNFα), lipopolysaccharide, and interleukin 1β (IL1β; Figure S5 and data not shown). TNFα and IL1β induced rapid upregulation of E-selectin with peak expression 6 hr post treatment in some but not all of the hiPSC-ECs examined. HUVECs exhibited robust upregulation of E-selectin upon TNFα and IL1β treatment, as expected. Furthermore, ICAM-1 upregulation in hiPSC-ECs was more prominent after 6 hr of TNFα treatment, and comparable with HUVECs. ICAM-1 was similarly induced in hiPSC-ECs and HUVECs 24 hr post treatment with either TNFα or IL1β. Upregulation of VCAM-1 was not observed in hiPSC-ECs, in contrast to HUVECs. All subsequent experiments were performed using TNFα, as it was the most potent pro-inflammatory agent in hiPSC-ECs. CD31+ and CD34+ hiPSC-ECs exhibited similar induction of E-selectin and ICAM-1 6 hr and 12 hr post stimulation, although this was lower than in HUVECs (Figures 5A–5D). The 12 hr time point was specifically chosen, as it was optimal for pre-stimulation of ECs for leukocyte adhesion studies. In order to investigate whether hiPSC-ECs can be used to study endothelial-leukocyte interactions, we established an assay to assess leukocyte adhesion under flow in a commercial system with eight parallel microchannels. hiPSC-ECs or primary ECs were seeded into the microfluidic channels and leukocyte perfusion was precisely controlled by a microfluidic pump. Adhesion of human leukocytes to ECs was investigated under flow at venous shear stress (0.5 dyn/cm^2). Leukocytes were perfused for 5 min, followed by additional perfusion for 5 min with culture medium to wash away all non-specifically attached cells. Pre-treatment of ECs with TNFα for 12 hr increased leukocyte adhesion significantly compared with non-treated ECs (Figure 5E and Video S1). CD31+ and CD34+ hiPSC-ECs were similar with respect to the numbers of adherent leukocytes per field, although HUVECs had significantly higher numbers (Figure 5F). These data showed that CD31+ and CD34+ hiPSC-ECs exhibit comparable inflammatory responses *in vitro*, and can potentially be used to study leukocyte cell interactions, although perhaps with less adhesion "strength" than HUVECs.

Comparison of Primary and hiPSC-ECs in an *In Vitro* Vasculogenesis Assay

We next examined the ability of CD31+ and CD34+ hiPSC-ECs to form a two-dimensional vascular plexus *in vitro* compared with primary ECs, as described previously (Evensen et al., 2009; Orlova et al., 2014a). We observed that hiPSC-ECs were more sensitive to the source of stromal cells than primary ECs. To identify the most reliable stromal cells to support hiPSC-EC sprouting *in vitro*, we screened several batches of CD31-cells from the differentiating hiPSC cultures (hiPSC-pericytes [hiPSC-Ps]; Orlova et al., 2014a), primary human bone marrow stromal cells (BMSCs), and human cardiac fibroblasts (huCFs). Somewhat unexpectedly, huCFs supported hiPSC-EC sprouting better than other stromal cells (Figures 6B, S6C, and S6D). By contrast, BMSCs were most potent in supporting sprouting of primary HUVECs and HDMECs compared with CD31-hiPSC-Ps (CD31-hiPSC-P) and huCFs (Figures S6A and S6B), although they supported sprouting of hiPSC-ECs poorly (Figures S6C and S6D). Therefore, huCFs were selected as the preferred stromal cell to compare hiPSC-ECs and primary ECs. In this assay, we thus co-cultured huCFs with CD31+ and CD34+ hiPSC-ECs, HUVECs, and HDMECs (Figures 6B–6D). Interestingly, under these conditions, CD31+ hiPSC-ECs formed very dense sprouting networks with total vessel lengths and numbers of junctions significantly higher than CD34+ hiPSC-ECs, HUVECs, or HDMECs (Figure 6D). CD34+ hiPSC-ECs were more similar to HUVECs and formed denser vascular networks than HDMECs, although these were less organized and had thinner sprouts compared with HUVECs. Since hiPSC-ECs exhibited embryonic-like characteristics and had a more prominent arterial-like phenotype, we also examined expression of the nuclear transcription factor SOX17. We found that SOX17 marked hiPSC-EC nuclei in the co-culture system, but not nuclei, of primary ECs (Figure 6C). Finally, independent batches of

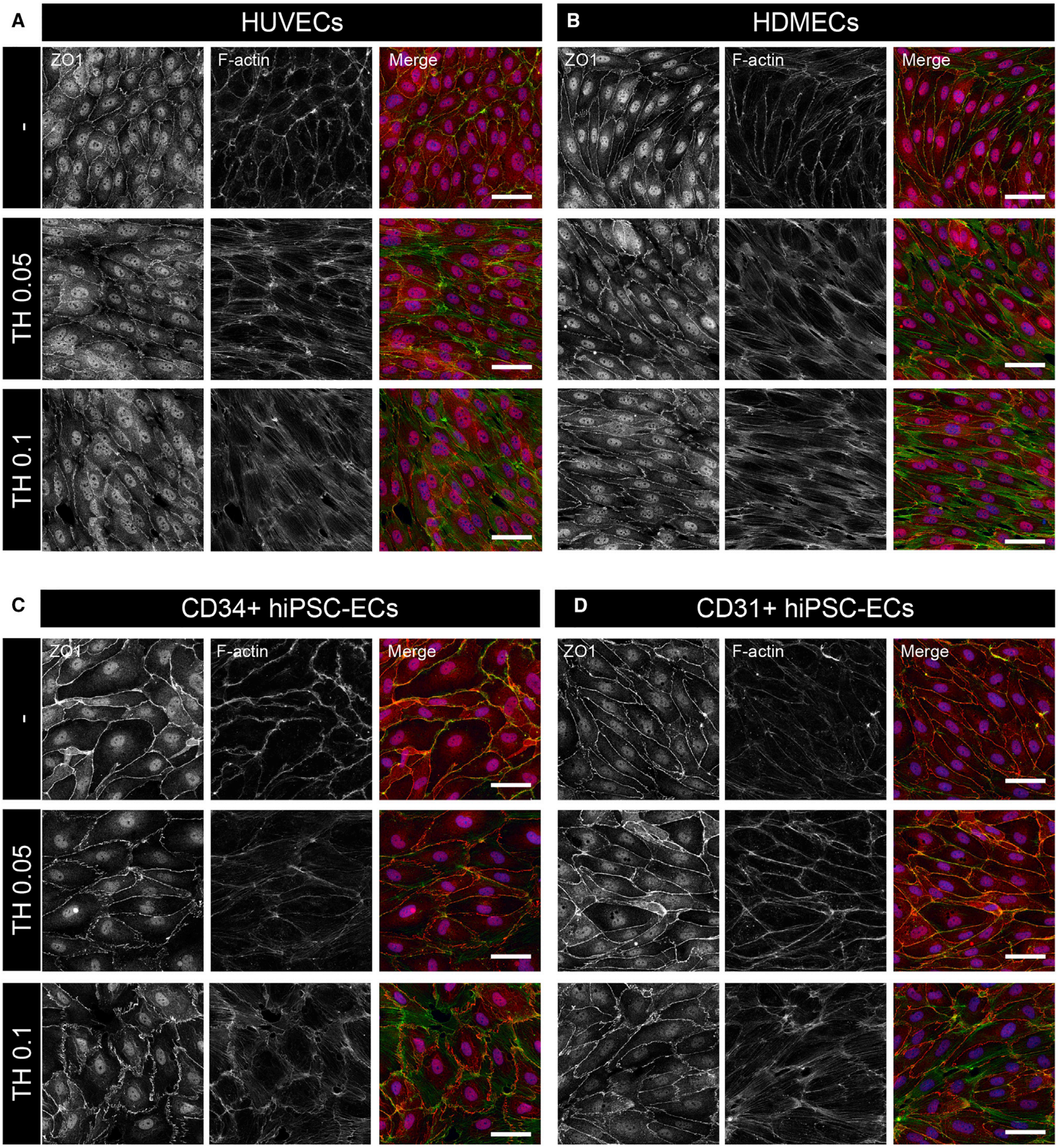

Figure 4. Comparison of Junctional Integrity in Primary and hiPSC-ECs

(A–D) Junctional integrity in primary cells and hiPSC-ECs was analyzed using tight junctional marker (ZO1) counterstained with F-actin in HUVECs (A), HDMECs (B), CD34+ hiPSC-ECs (C), and CD31+ hiPSC-ECs (D) upon control stimulation with medium only (–) or thrombin (TH; 0.05 U/mL and 0.1 U/mL) for 30 min. Disassembly of cell junctions and reorganization of cortical actin and actin stress fiber formation was observed in HUVECs and HDMECs upon thrombin (0.05 U/mL and 0.1 U/mL) stimulation. CD34+ hiPSC-ECs and CD31+ hiPSC-ECs showed robust response upon thrombin (0.1 U/mL) stimulation. Adherents junctions visualized with VEC and counterstained with F-actin are shown in Figure S4. Representative pictures are shown from experiments performed with three batches of CD31+ and CD34+ hiPSC-ECs, HUVECs from three batches (two donors, and two independent batches for one of the donors), and for HDMECs a single donor. Scale bar represents 50 μm.

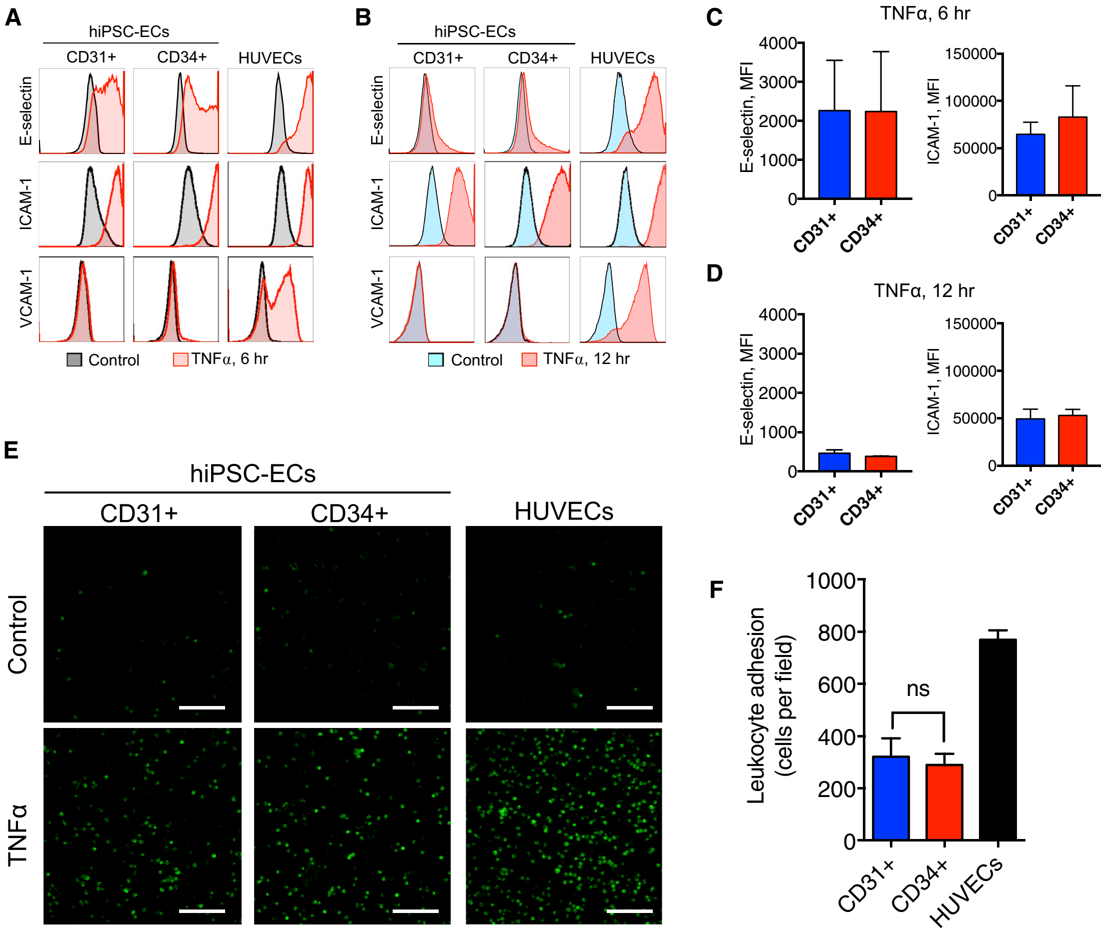

Figure 5. Comparison of Inflammatory Responses in Primary and hiPSC-ECs

(A) FACS analysis of surface expression of E-selectin, ICAM-1, and VCAM-1 in untreated cells (black filled histograms) or after 6 hr of treatment (red filled histograms) with TNFα (10 ng/mL).

(B) FACS analysis of surface expression of E-selectin, ICAM-1, and VCAM-1 in untreated cells (blue filled histograms) or after 12 hr of treatment (red filled histograms) with TNFα (10 ng/mL).

(C) Quantification of surface expression of E-selectin and ICAM-1 on CD34+ and CD31+ after 6 hr of treatment with TNFα (10 ng/mL). Error bars are shown as ±SD of three independent biological experiments.

(D) Quantification of surface expression of E-selectin and ICAM-1 on CD34+ and CD31 + after 12 hr of treatment with TNFα (10 ng/mL). Error bars are shown as ±SD of three independent biological experiments.

(E) Assessment of leukocyte adhesion under flow. Representative images of adhesion of leukocytes (green) to non-treated (control) or TNFα-treated (12 hr, 10 ng/mL) CD31+ and CD34+ hiPSC-ECs, and HUVEC. Scale bar represents 250 μm.

(F) Quantification of leukocyte adhesion per field to TNFα-treated CD31+ and CD34+ hiPSC-ECs, and HUVEC. Data are shown as ±SD (CD31 +, n = 5; CD34 +, n = 4; HUVEC, n = 2); ns, not significant.

CD31+ and CD34+ hiPSC-ECs were very similar, in agreement with our previous results (Figure 6D).

Comparison of Primary and hiPSC-ECs in an *In Vivo* Vasculogenesis Assay

We next tested the *in vivo* functionality of hiPSC-ECs and their ability to form functional, perfused vessels in a heterotopic *in vivo* differentiation assay, described previously (Sacchetti et al., 2016). We first examined the potential of CD31+ hiPSC-ECs co-transplanted with BMSCs to integrate into vessels *in vivo*. CD31+ hiPSC-ECs were mixed with BMSCs and growth factor-reduced Matrigel in different ratios: 1 million hiPSC-ECs and 1 million BMSCs (1:1), 2 million hiPSC-ECs with 1 million BMSC (2:1), and *vice versa* (1:2). Formation of vascular networks containing red blood cells, indirectly suggesting vascular perfusion, was observed at all cell ratios tested (Figure S7), although, overall, the 2:1 ratio gave the best result and was similar

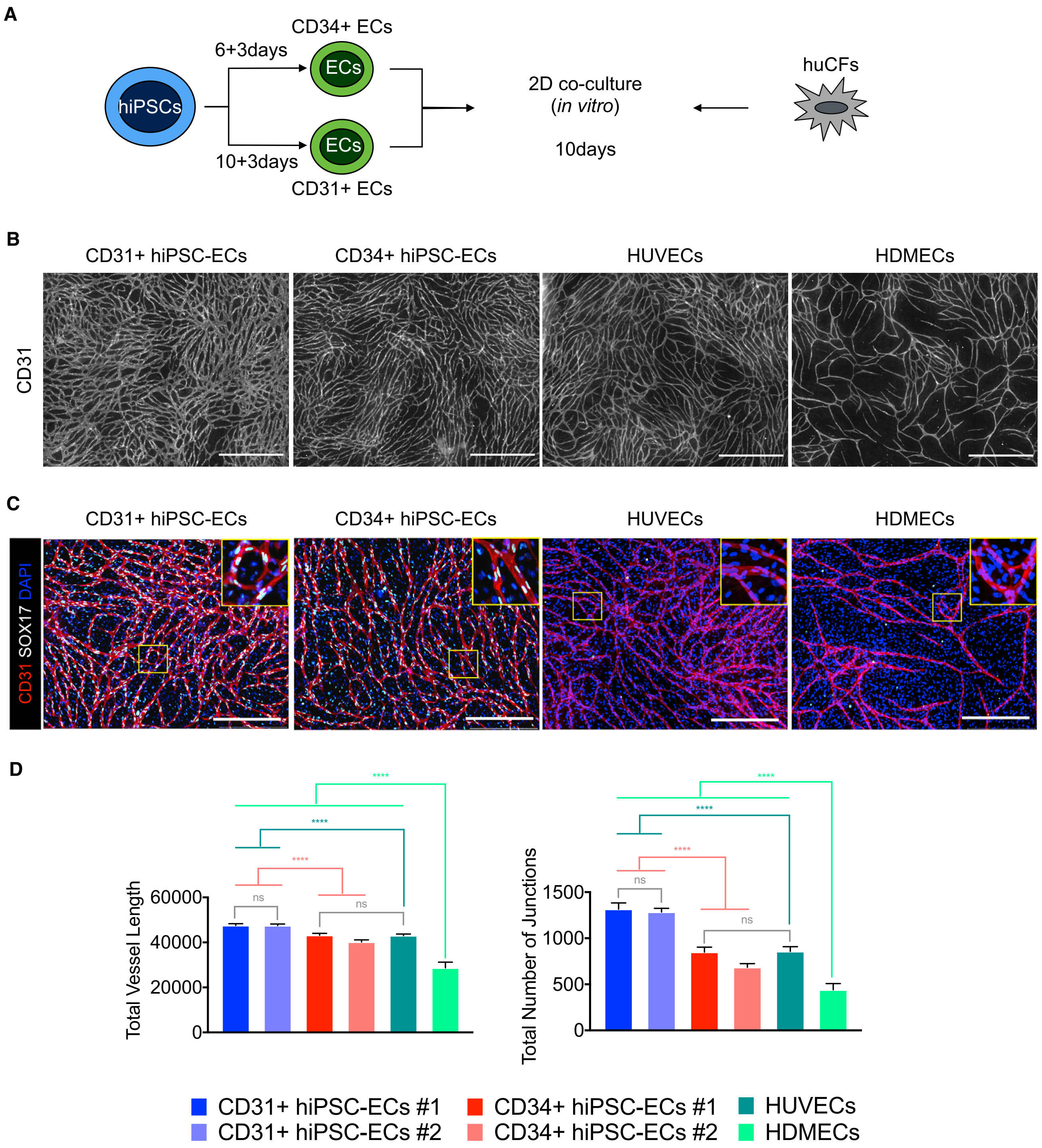

Figure 6. Comparison of Primary and hiPSC-ECs in an *In Vitro* Vasculogenesis Assay

(A) Schematic representation of an *in vitro* vasculogenesis assay. hiPSC-derived CD31 + or CD34 + cells are combined with stromal cells (huCFs). The cells are mixed plated into 96-well plates and the EC sprouting network is visualized 10 days after co-culture.

(B) Representative immunofluorescent images of an *in vitro* vasculogenesis sprouting assay at day 10 of the co-culture used for quantification of the sprouting network. ECs are visualized with anti-CD31 (white). Automatically stitched images (10× objective, 4 × 4 focus planes) are shown. The images were taken with an automated imaging system with autofocus on CD31. Scale bar represents 1,000 μm.

(legend continued on next page)

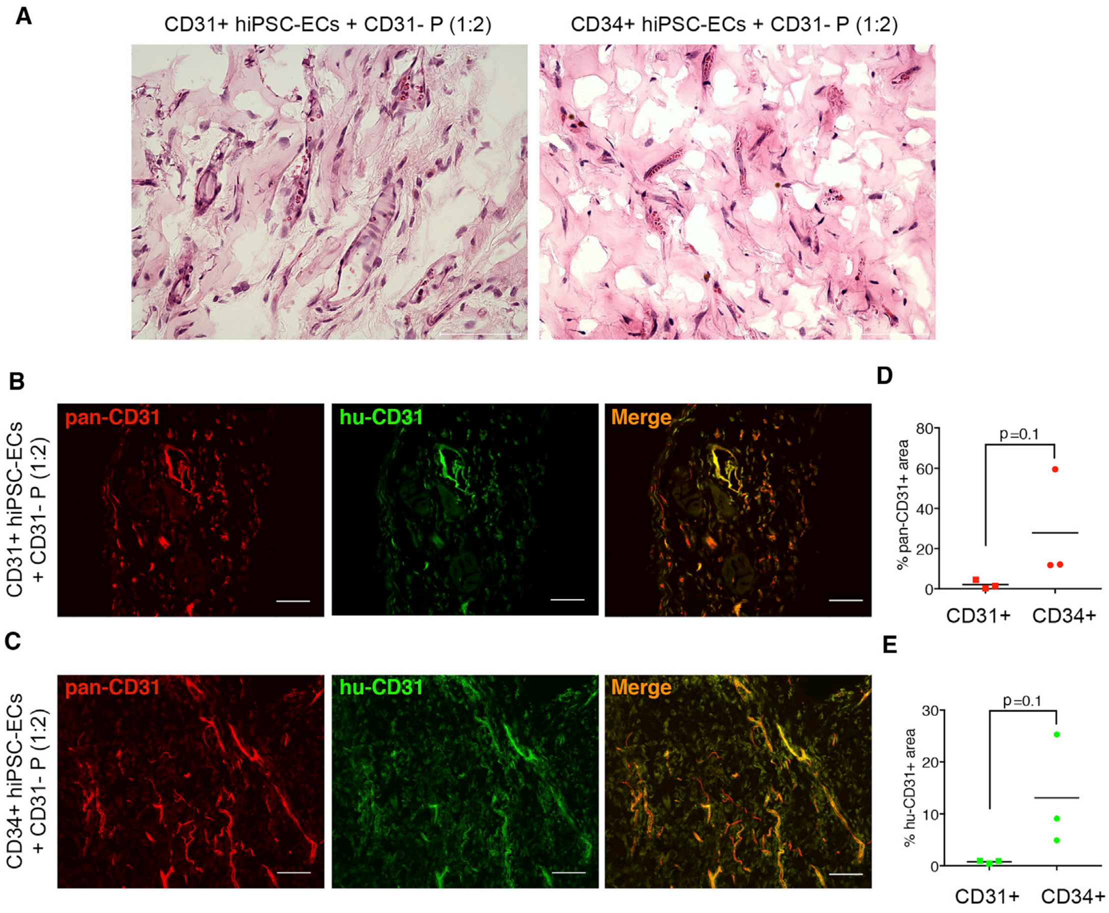

Figure 7. Comparison of CD31+ and CD34+ hiPSC-ECs in an *In Vivo* Vasculogenesis Assay

(A) H&E images of Matrigel plugs. Representative images of Matrigel plugs with CD31+ hiPSC-ECs and CD34+ hiPSC-ECs co-transplanted with CD31-hiPSC-Ps (1:2). Scale bar represents 75 μm.

(B and C) Representative images of Matrigel plugs with CD31+ hiPSC-ECs and CD34+ hiPSC-ECs co-transplanted with CD31-hiPSC-Ps. IHC with pan-specific (red) and anti-human (green) CD31 antibody or overlay (orange). Scale bar represents 100 μm.

(D) Quantification of vascular density using pan-specific CD31 (pan-CD31) in Matrigel plugs CD31+ hiPSC-ECs and CD34+ hiPSC-ECs co-transplanted with CD31-hiPSC-Ps (n = 3).

(E) Quantification of vascular density using human-specific CD31 (hu-CD31) in Matrigel plugs CD31+ hiPSC-ECs and CD34+ hiPSC-ECs co-transplanted with CD31-hiPSC-Ps (n = 3).

to Matrigel transplants containing a 1:1 ratio of HUVECs and BMSCs. We next compared the *in vivo* potential of CD34+ and CD31+ hiPSC-ECs (2 million cells) co-transplanted with CD31-hiPSC-P (1 million) (2:1 ratio), derived as described previously (Orlova et al., 2014b). Interestingly, both CD31+ and CD34+ hiPSC-ECs formed perfused vascular networks, as indirectly suggested by the presence of red blood cells on immunohistochemistry (IHC) sections (Figure 7A). The presence of human ECs was confirmed with human-specific and pan-specific (human and mouse) antibody against CD31 (Figures 7B and 7C). Vascular density appeared higher in the Matrigel plugs containing CD34 + cells compared with CD31 + cells, although this was not statistically significant (Figures 7D and 7E). Therefore, we concluded that both CD31+ and CD34+ hiPSC-ECs can form functional blood vessels *in vivo* although

(C) Representative immunofluorescent images of an *in vitro* vasculogenesis sprouting assay at day 10 of the co-culture. ECs are visualized with anti-CD31 (red), SOX17 (white), and DAPI (blue). Higher magnification is shown in the framed area. Scale bar represents 500 μm.

(D) Quantification of EC sprouting network at day 10 of the co-culture. Quantification was performed with Angiotool software. The total vessel length and total number of junctions are shown. Automatically stitched images (10× objective, 4 × 4 focus planes) from six co-cultures were used for quantification. Data are shown as ±SD. ****$p < 0.0001$.

the transplantation conditions and stromal cell source might need further optimization when comparing with primary ECs.

DISCUSSION

Since the initial discovery of hiPSCs, directed differentiation protocols to form specific cell types in defined conditions have significantly improved. With regard to ECs, many protocols have been developed that result in fairly high percentages of ECs that vary from 30% to 80% of the differentiated cell population (Bao et al., 2015; Patsch et al., 2015; Zhang et al., 2017). Furthermore, defined matrices, such as recombinant vitronectin and laminin, have also been used (Nguyen et al., 2016; Zhang et al., 2017). ECs can be purified and conveniently cryopreserved for immediate use after thaw in various functional assays (Orlova et al., 2014b). In the present study, we carried out functional assays on hiPSC-ECs at the same passage 2 (P2), which made the biological replicates highly comparable without the need for internal normalization within the assay, as demonstrated. Nevertheless, despite their potential utility, primary ECs are still preferred to hiPSC-ECs in vascular research and assays, likely due to apparent differences in their developmental and differentiation states. hiPSC-ECs are indeed more similar to embryonic ECs, based on their marker and gene expression profiles (Orlova et al., 2014a; Rufaihah et al., 2013; Vazão et al., 2017). However, this can have advantages for certain applications, such as screening for embryonic vascular toxicity (Vazão et al., 2017), and perhaps modeling tumor vasculature, since it is also considered immature. Recent work by our group and others has focused on differentiating hiPSC to ECs of the more prominent vascular beds and tissue-specific ECs, such as arterial, venous, and cardiac ECs, as well as so-called EC colony-forming cells (Giacomelli et al., 2017; Ng et al., 2016; Palpant et al., 2017; Prasain et al., 2014; Zhang et al., 2017). Taken together, these findings contribute to enhancing the value of hiPSC-ECs in imminent applications such as drug discovery and regenerative medicine. However, understanding exactly how hiPSC-ECs are similar to or differ from primary ECs through side-by-side comparisons in standard assays is essential for their wider acceptance. An important first step is to identify conditions that support both primary and hiPSC-ECs. This is preferably based on defined cell culture growth medium; synthetic matrices (Nguyen et al., 2017); and, as necessary, common stromal cell types in co-culture for vasculogenesis assays. Several groups have investigated the impact of the developmental origin of pericytes and smooth muscle cells on vasculogenesis by HUVECs (Bargehr et al., 2016; Kumar et al., 2017). Here, we examined the interaction of hiPSC-ECs with stromal cells and report that they have much more stringent stromal cell requirements. For instance, BMSCs were very poor in supporting of sprouting of hiPSC-ECs compared with HUVECs *in vitro* and to a lesser extent *in vivo*, and different stromal cell to EC ratios might be required for efficient vascularization. Although differences in interaction of hiPSC-ECs and primary ECs with the stromal cells have not been addressed here, this would be of interest in future studies.

Comparison of barrier function and inflammatory responses between ECs differentiated from independent isogenic and non-isogenic (non-integrating/DNA-free) hiPSC lines revealed high similarity between independent EC batches. This demonstrates that hiPSCs are a highly consistent source of donor-specific ECs so that genetically induced changes in these features might be regarded as disease-specific phenotypes even in the absence of an isogenic control. Although we found that CD31+ hiPSC-ECs isolated at day 10 of differentiation were more similar to each other than early CD34+ hiPSC-ECs isolated at day 6, this could be due to slight differences in (dynamic) differentiation states, and variable delays less prominent on day 10. Therefore, despite a shorter differentiation protocol and the highly proliferative state of CD34+ hiPSC-ECs, the longer protocol would be preferred for producing more robust batches of ECs for disease modeling purposes. In addition, examination of barrier function across a wide set of hiPSC-ECs revealed that CD31+ hiPSC-ECs had tighter barriers than either CD34+ hiPSC-ECs or primary ECs, like HUVECs and HDMECs. Unexpectedly, hiPSC-ECs did not respond to histamine, a known barrier-disrupting compound. These data contrast with those previously for hiPSC-ECs (Adams et al., 2013) but were highly consistent between all lines here. However, there was a difference in the timing of barrier reduction: Adams et al. (2013) showed a delayed response approximately 30 min to 1 hr post stimulation, which also contrasts with reports of other groups for histamine-mediated decreases in endothelial barrier function (Aman et al., 2012; Szulcek et al., 2014; van Nieuw Amerongen et al., 1998). However, both CD31+ and CD34+ hiPSC-ECs did show a pronounced response to relatively low doses of thrombin (0.1 U/mL), with barrier function significantly and non-reversibly altered. The thrombin concentration used here was also significantly lower compared with a previous report, where 20 U/mL was used (Patsch et al., 2015). This may have been dictated by different culture and stimulation conditions but our specific aim was to carry out the assays as would normally be done using primary ECs where both 0.05 and 0.1 U/mL thrombin are reportedly sufficient for barrier disruption. In addition to rapid barrier-disrupting agents (histamine and thrombin), we also found that hiPSC-ECs were very sensitive to VEGF, which resulted in a

pronounced decrease in the barrier in all hiPSC-ECs examined. Furthermore, no significant difference was found in migration rates between CD31+ and CD34+ hiPSC-ECs.

Examination of inflammatory responses further revealed that both CD31+ and CD34+ hiPSC-ECs responded to TNFα in a similar manner to HUVECs and were capable of upregulating major pro-inflammatory adhesive receptors, such as E-selectin and ICAM-1. However, no upregulation of VCAM-1 was observed in any hiPSC-ECs examined, in contrast to primary ECs. These data also differ from previous reports (Adams et al., 2013; Patsch et al., 2015; Vazão et al., 2017). Primary ECs were also shown to exhibit differential upregulation of VCAM-1, much like ECs from different organs such as different compartments of the kidney vasculature, where there is prominent VCAM-1 expression in arteriolar endothelium but not in glomerular endothelium (Asgeirsdottir et al., 2012; Scott et al., 2013). Further examination of leukocyte adhesion under physiological flow revealed that CD31+ and CD34+ hiPSC-ECs were comparable, although less pro-adhesive, than HUVECs. Any inconsistences between ECs could be due to the developmental and tissue identity.

Overall, hiPSC-ECs have a number of advantages as model systems over primary ECs: (1) the possibility to derive large batches with very high numbers of high quality ECs from the same donor, all with the similar features to primary ECs; (2) high barrier functions compared with other peripheral ECs; and (3) inflammatory responses in which ECs and monocytes can be derived (isogenically) from the same donor. Present hurdles for hiPSC-ECs compared with primary ECs include (1) lower expression of pro-inflammatory adhesive receptors, such as E-selectin and lack of VCAM-1 induction; and (2) limited maturity (for instance, with lower expression of vWF, which might be a shortcoming in modeling certain genetic conditions). In the future, we expect the functional assays we have described will be useful in comparing hiPSC-ECs from more advanced differentiation protocols in which cells have more prominent venous- or tissue-specific identities, important in modeling genetic and other diseases associated with particular vascular beds.

In summary, we have provided here comprehensive characterization and line-to-line and batch-to-batch comparisons of hiPSC-ECs. We demonstrated that barrier function and inflammatory responses are highly consistent between different healthy hiPSC-EC lines, and therefore can be considered as a benchmark for standardization of functionality across different lines.

EXPERIMENTAL PROCEDURES

Details are provided in Supplemental Experimental Procedures.

hiPSC Lines and Maintenance

The following SeV reprogrammed hiPSCs lines were used in this study: FiPSC line generated from fibroblast (FiPSC line LUMC0020iCTRL), as described previously (Zhang et al., 2014), and hiPSCs from urine-derived cells (UiPSC lines): LUMC0054iCTRL (additional information available in public databases: http://hpscreg.eu/cell-line/LUMCi001-A and http://hpscreg.eu/cell-line/LUMCi001-A-1). hiPSCs were cultured on Matrigel-coated plates in mTeSR-1 or recombinant vitronectin-coated plates in TeSR-E8, all from STEMCELL Technologies, according to the manufacturer's instructions.

Differentiation of hiPSCs toward ECs

hiPSCs were maintained in mTeSR-1 or mTeSR-E8 and differentiated toward ECs using previously published protocols (Orlova et al., 2014b, 2014a).

Characterization of CD34+ and CD31+ hiPSC-ECs

Basic characterization of hiPSC-ECs, such as FACS analysis, immunofluorescence, and gene expression analyses, was performed as previously described (Orlova et al., 2014b).

Assessment of hiPSC-EC Functionality in an *In Vivo* Vasculogenesis Assay

The Matrigel plug assay was performed as previously described (Sacchetti et al., 2016). Experiments with hiPSC-ECs and BMSCs were carried out in compliance with relevant Italian laws and institutional guidelines for animals and all procedures were Institutional Animal Care and Use Committee approved. Experiments with hiPSC-ECs and CD31-hiPSC-P were approved by the Leiden University Medical Center animal experimental committee and the Commission Biotechnology in Animals of the Dutch Ministry of Agriculture.

Statistical Analysis

Statistical analyses were conducted with GraphPad Prism 7 software. One-way ANOVA with Tukey's multiple comparison for the analysis of three or more groups or Mann-Whitney test for analysis of two groups were used. The data are reported as mean ± SD.

SUPPLEMENTAL INFORMATION

Supplemental Information includes Supplemental Experimental Procedures, seven figures, one table, and one video and can be found with this article online at https://doi.org/10.1016/j.stemcr.2018.03.012.

AUTHOR CONTRIBUTIONS

O.V.H. performed and quantified ECIS experiments, C.F. established SeV reprogramming, F.v.d.H. performed differentiation of CD31+ and CD34+ hiPSC-ECs, D.C.F.S. and M.R. performed Matrigel plug assay, C.L.M. edited the manuscript, and V.V.O. designed the research, established and performed ECIS and flow experiments, quantified results, analyzed the data, and wrote the manuscript.

ACKNOWLEDGMENTS

The authors would like to acknowledge Ana Melo Bernardo for help with quantification of the endothelial-leukocyte interaction assay and Mahito Nakanishi for provision of SeV. The work was supported by the European Community's Seventh Framework Programme (FP7/2007-2013 under 602423) and the European Union's Horizon 2020 Framework Programme (668724).

Received: September 13, 2017
Revised: March 12, 2018
Accepted: March 13, 2018
Published: April 12, 2018

REFERENCES

Adams, W.J., Zhang, Y., Cloutier, J., Kuchimanchi, P., Newton, G., Sehrawat, S., Aird, W.C., Mayadas, T.N., Luscinskas, F.W., and García-Cardeña, G. (2013). Functional vascular endothelium derived from human induced pluripotent stem cells. Stem Cell Reports *1*, 105–113.

Aird, W.C. (2012). Endothelial cell heterogeneity. Cold Spring Harb. Perspect. Med. *2*, a006429.

Aman, J., van Bezu, J., Damanafshan, A., Huveneers, S., Eringa, E.C., Vogel, S.M., Groeneveld, A.B., Vonk Noordegraaf, A., van Hinsbergh, V.W., and van Nieuw Amerongen, G.P. (2012). Effective treatment of edema and endothelial barrier dysfunction with imatinib. Circulation *126*, 2728–2738.

Asgeirsdottir, S.A., van Solingen, C., Kurniati, N.F., Zwiers, P.J., Heeringa, P., van Meurs, M., Satchell, S.C., Saleem, M.A., Mathieson, P.W., Banas, B., et al. (2012). MicroRNA-126 contributes to renal microvascular heterogeneity of VCAM-1 protein expression in acute inflammation. Am. J. Physiol. Renal Physiol. *302*, F1630–F1639.

Bao, X., Lian, X., Dunn, K.K., Shi, M., Han, T., Qian, T., Bhute, V.J., Canfield, S.G., and Palecek, S.P. (2015). Chemically-defined albumin-free differentiation of human pluripotent stem cells to endothelial progenitor cells. Stem Cell Res. *15*, 122–129.

Bargehr, J., Low, L., Cheung, C., Bernard, W.G., Iyer, D., Bennett, M.R., Gambardella, L., and Sinha, S. (2016). Embryological origin of human smooth muscle cells influences their ability to support endothelial network formation. Stem Cells Transl. Med. *5*, 946–959.

Cai, J., Orlova, V.V., Cai, X., Eekhoff, E.M.W., Zhang, K., Pei, D., Pan, G., Mummery, C.L., and Ten Dijke, P. (2015). Induced pluripotent stem cells to model human fibrodysplasia ossificans progressiva. Stem Cell Reports *5*, 963–970.

Chen, I.P., Fukuda, K., Fusaki, N., Iida, A., Hasegawa, M., Lichtler, A., and Reichenberger, E.J. (2013). Induced pluripotent stem cell reprogramming by integration-free Sendai virus vectors from peripheral blood of patients with craniometaphyseal dysplasia. Cell. Reprogram. *15*, 503–513.

Evensen, L., Micklem, D.R., Blois, A., Berge, S.V., Aarsæther, N., Littlewood-Evans, A., Wood, J., and Lorens, J.B. (2009). Mural cell associated VEGF is required for organotypic vessel formation. PLoS One *4*, e5798.

Fusaki, N., Ban, H., Nishiyama, A., Saeki, K., and Hasegawa, M. (2009). Efficient induction of transgene-free human pluripotent stem cells using a vector based on Sendai virus, an RNA virus that does not integrate into the host genome. Proc. Jpn. Acad. Ser. B Phys. Biol. Sci. *85*, 348–362.

Giacomelli, E., Bellin, M., Sala, L., van Meer, B.J., Tertoolen, L.G.J., Orlova, V.V., and Mummery, C.L. (2017). Three-dimensional cardiac microtissues composed of cardiomyocytes and endothelial cells co-differentiated from human pluripotent stem cells. Development https://doi.org/10.1242/dev.143438.

Giannotta, M., Trani, M., and Dejana, E. (2013). VE-cadherin and endothelial adherens junctions: active guardians of vascular integrity. Dev. Cell *26*, 441–454.

Hajishengallis, G., and Chavakis, T. (2013). Endogenous modulators of inflammatory cell recruitment. Trends Immunol. *34*, 1–6.

Hildebrand, L., Rossbach, B., Kühnen, P., Gossen, M., Kurtz, A., Reinke, P., Seemann, P., and Stachelscheid, H. (2016). Generation of integration free induced pluripotent stem cells from fibrodysplasia ossificans progressiva (FOP) patients from urine samples. Stem Cell Res. *16*, 54–58.

Iwata, Y., Klaren, W.D., Lebakken, C.S., Grimm, F.A., and Rusyn, I. (2017). High-content assay multiplexing for vascular toxicity screening in induced pluripotent stem cell-derived endothelial cells and human umbilical vein endothelial cells. Assay Drug Dev. Technol. https://doi.org/10.1089/adt.2017.786.

Komarova, Y.A., Kruse, K., Mehta, D., and Malik, A.B. (2017). Protein interactions at endothelial junctions and signaling mechanisms regulating endothelial permeability. Circ. Res. *120*, 179–206.

Kumar, A., D'Souza, S.S., Moskvin, O.V., Toh, H., Wang, B., Zhang, J., Swanson, S., Guo, L.W., Thomson, J.A., and Slukvin, I.I. (2017). Specification and diversification of pericytes and smooth muscle cells from mesenchymoangioblasts. Cell Rep. *19*, 1902–1916.

Ng, E.S., Azzola, L., Bruveris, F.F., Calvanese, V., Phipson, B., Vlahos, K., Hirst, C., Jokubaitis, V.J., Yu, Q.C., Maksimovic, J., et al. (2016). Differentiation of human embryonic stem cells to HOXA+ hemogenic vasculature that resembles the aorta-gonad-mesonephros. Nat. Biotechnol. *34*, 1168–1179.

Nguyen, E.H., Daly, W.T., Le, N.N.T., Farnoodian, M., Belair, D.G., Schwartz, M.P., Lebakken, C.S., Ananiev, G.E., Saghiri, M.A., Knudsen, T.B., et al. (2017). Versatile synthetic alternatives to Matrigel for vascular toxicity screening and stem cell expansion. Nat. Biomed. Eng. *1*. https://doi.org/10.1038/s41551-017-0096.

Nguyen, M.T.X., Okina, E., Chai, X., Tan, K.H., Hovatta, O., Ghosh, S., and Tryggvason, K. (2016). Differentiation of human embryonic stem cells to endothelial progenitor cells on laminins in defined and xeno-free systems. Stem Cell Reports *7*, 802–816.

Nishimura, K., Sano, M., Ohtaka, M., Furuta, B., Umemura, Y., Nakajima, Y., Ikehara, Y., Kobayashi, T., Segawa, H., Takayasu, S., et al. (2011). Development of defective and persistent Sendai virus vector: a unique gene delivery/expression system ideal for cell reprogramming. J. Biol. Chem. *286*, 4760–4771.

Nourshargh, S., and Alon, R. (2014). Leukocyte migration into inflamed tissues. Immunity *41*, 694–707.

Ono, M., Hamada, Y., Horiuchi, Y., Matsuo-Takasaki, M., Imoto, Y., Satomi, K., Arinami, T., Hasegawa, M., Fujioka, T., Nakamura, Y.,

and Noguchi, E. (2012). Generation of induced pluripotent stem cells from human nasal epithelial cells using a Sendai virus vector. PLoS One *7*, e42855.

Orlova, V.V., Drabsch, Y., Freund, C., Petrus-Reurer, S., van den Hil, F.E., Muenthaisong, S., Dijke, P.T., and Mummery, C.L. (2014a). Functionality of endothelial cells and pericytes from human pluripotent stem cells demonstrated in cultured vascular plexus and zebrafish xenografts. Arterioscler. Thromb. Vasc. Biol. *34*, 177–186.

Orlova, V.V., van den Hil, F.E., Petrus-Reurer, S., Drabsch, Y., Ten Dijke, P., and Mummery, C.L. (2014b). Generation, expansion and functional analysis of endothelial cells and pericytes derived from human pluripotent stem cells. Nat. Protoc. *9*, 1514–1531.

Palpant, N.J., Pabon, L., Friedman, C.E., Roberts, M., Hadland, B., Zaunbrecher, R.J., Bernstein, I., Zheng, Y., and Murry, C.E. (2017). Generating high-purity cardiac and endothelial derivatives from patterned mesoderm using human pluripotent stem cells. Nat. Protoc. *12*, 15–31.

Passier, R., Orlova, V., and Mummery, C. (2016). Complex tissue and disease modeling using hiPSCs. Cell Stem Cell *18*, 309–321.

Patsch, C., Challet-Meylan, L., Thoma, E.C., Urich, E., Heckel, T., O'Sullivan, J.F., Grainger, S.J., Kapp, F.G., Sun, L., Christensen, K., et al. (2015). Generation of vascular endothelial and smooth muscle cells from human pluripotent stem cells. Nat. Cell Biol. https://doi.org/10.1038/ncb3205.

Prasain, N., Lee, M.R., Vemula, S., Meador, J.L., Yoshimoto, M., Ferkowicz, M.J., Fett, A., Gupta, M., Rapp, B.M., Saadatzadeh, M.R., et al. (2014). Differentiation of human pluripotent stem cells to cells similar to cord-blood endothelial colony–forming cells. Nat. Biotechnol. *32*, 1151–1157.

Rufaihah, A.J., Huang, N.F., Kim, J., Herold, J., Volz, K.S., Park, T.S., Lee, J.C., Zambidis, E.T., Reijo-Pera, R., and Cooke, J.P. (2013). Human induced pluripotent stem cell-derived endothelial cells exhibit functional heterogeneity. Am. J. Transl. Res. *5*, 21–35.

Sacchetti, B., Funari, A., Remoli, C., Giannicola, G., Kogler, G., Liedtke, S., Cossu, G., Serafini, M., Sampaolesi, M., Tagliafico, E., et al. (2016). No identical "mesenchymal stem cells" at different times and sites: human committed progenitors of distinct origin and differentiation potential are incorporated as adventitial cells in microvessels. Stem Cell Reports *6*, 897–913.

Samuel, R., Duda, D.G., Fukumura, D., and Jain, R.K. (2015). Vascular diseases await translation of blood vessels engineered from stem cells. Sci. Transl. Med. *7*, 309rv6.

Scott, D.W., Vallejo, M.O., and Patel, R.P. (2013). Heterogenic endothelial responses to inflammation: role for differential N-glycosylation and vascular bed of origin. J. Am. Heart Assoc. *2*, e000263.

Shi, Y., Inoue, H., Wu, J.C., and Yamanaka, S. (2016). Induced pluripotent stem cell technology: a decade of progress. Nat. Rev. Drug Discov. *16*. https://doi.org/10.1038/nrd.2016.245.

Stolwijk, J.A., Matrougui, K., Renken, C.W., and Trebak, M. (2014). Impedance analysis of GPCR-mediated changes in endothelial barrier function: overview and fundamental considerations for stable and reproducible measurements. Pflugers Arch. https://doi.org/10.1007/s00424-014-1674-0.

Szulcek, R., Bogaard, H.J., and van Nieuw Amerongen, G.P. (2014). Electric cell-substrate impedance sensing for the quantification of endothelial proliferation, barrier function, and motility. J. Vis. Exp. https://doi.org/10.3791/51300.

van Nieuw Amerongen, G.P., Draijer, R., Vermeer, M.A., and van Hinsbergh, V.W. (1998). Transient and prolonged increase in endothelial permeability induced by histamine and thrombin: role of protein kinases, calcium, and RhoA. Circ. Res. *83*, 1115–1123.

Vazão, H., Rosa, S., Barata, T., Costa, R., Pitrez, P.R., Honório, I., de Vries, M.R., Papatsenko, D., Benedito, R., Saris, D., et al. (2017). High-throughput identification of small molecules that affect human embryonic vascular development. Proc. Natl. Acad. Sci. USA *114*, E3022–E3031.

Zhang, J., Chu, L.-F., Hou, Z., Schwartz, M.P., Hacker, T., Vickerman, V., Swanson, S., Leng, N., Nguyen, B.K., Elwell, A., et al. (2017). Functional characterization of human pluripotent stem cell-derived arterial endothelial cells. Proc. Natl. Acad. Sci. USA *114*, E6072–E6078.

Zhang, M., D'Aniello, C., Verkerk, A.O., Wrobel, E., Frank, S., Ward-van Oostwaard, D., Piccini, I., Freund, C., Rao, J., Seebohm, G., et al. (2014). Recessive cardiac phenotypes in induced pluripotent stem cell models of Jervell and Lange-Nielsen syndrome: disease mechanisms and pharmacological rescue. Proc. Natl. Acad. Sci. USA *111*, E5383–E5392.

Stem Cell Reports, Volume *10*

Supplemental Information

Inflammatory Responses and Barrier Function of Endothelial Cells Derived from Human Induced Pluripotent Stem Cells

Oleh V. Halaidych, Christian Freund, Francijna van den Hil, Daniela C.F. Salvatori, Mara Riminucci, Christine L. Mummery, and Valeria V. Orlova

Supplemental Figures and Legends

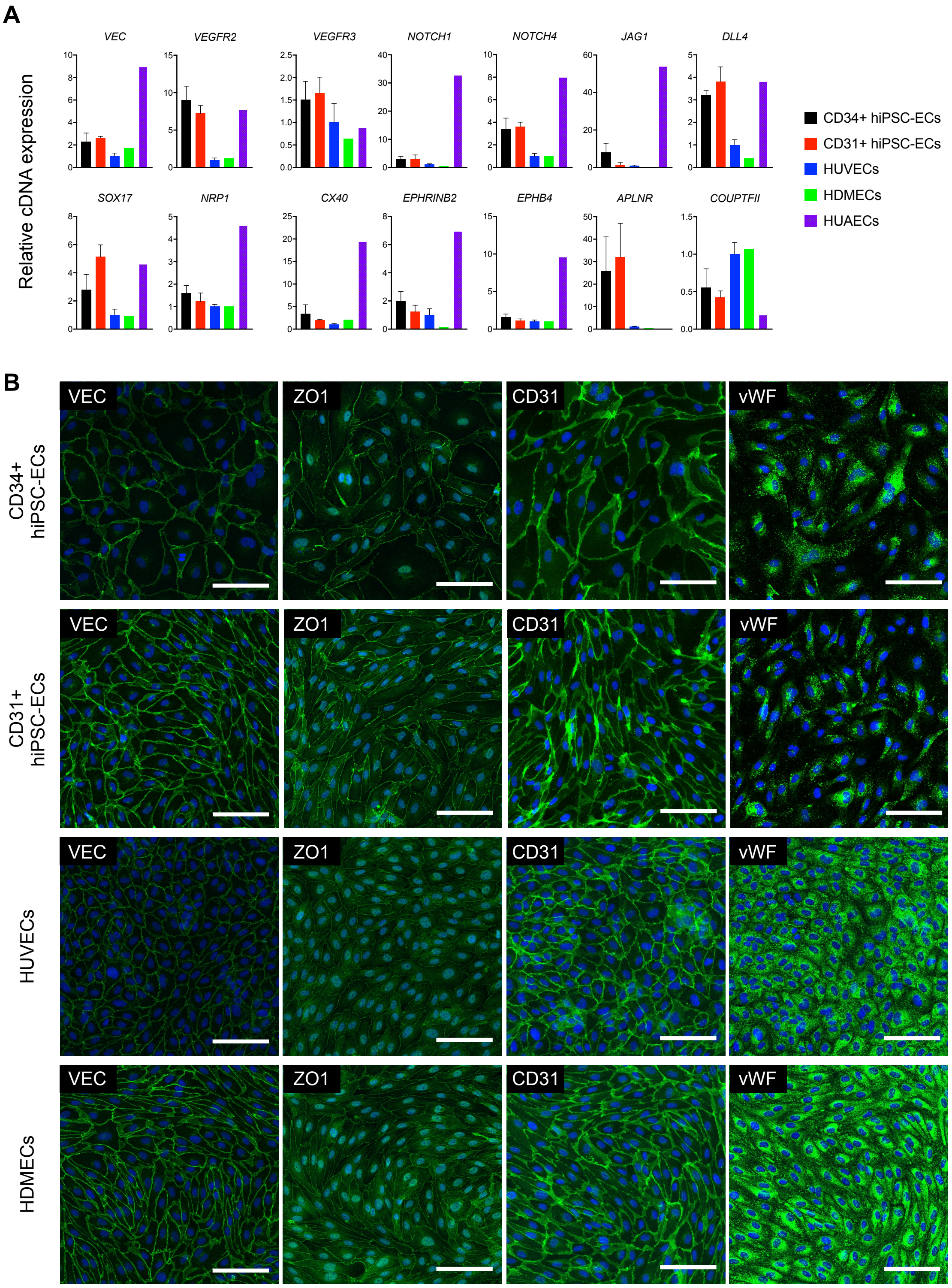

Figure S1. Related to Figure 1. Comparison of hiPSC-derived and primary ECs. (A) Gene expression analysis of expression of arterial and venous markers in isolated CD34+ and CD31+ hiPSC-ECs at passage 2 (P2) and primary ECs (HUVECs, HDMECs and HUAECs). Average values for three batches of CD31+ and CD34+ hiPSC-ECs, HUVECs from three batches (two donors, and two independent batches for one of the donors), HDMECs and HUAECs from a single donor are shown. Error bars are ±SD. (B) Immunofluorescent analysis of EC markers VEC, ZO1, CD31 and vWF on isolated CD34+ and CD31+ hiPSC-ECs (P2) and primary ECs (HUVECs and HDMECs)(P4-P5). Scale bar 100μm.

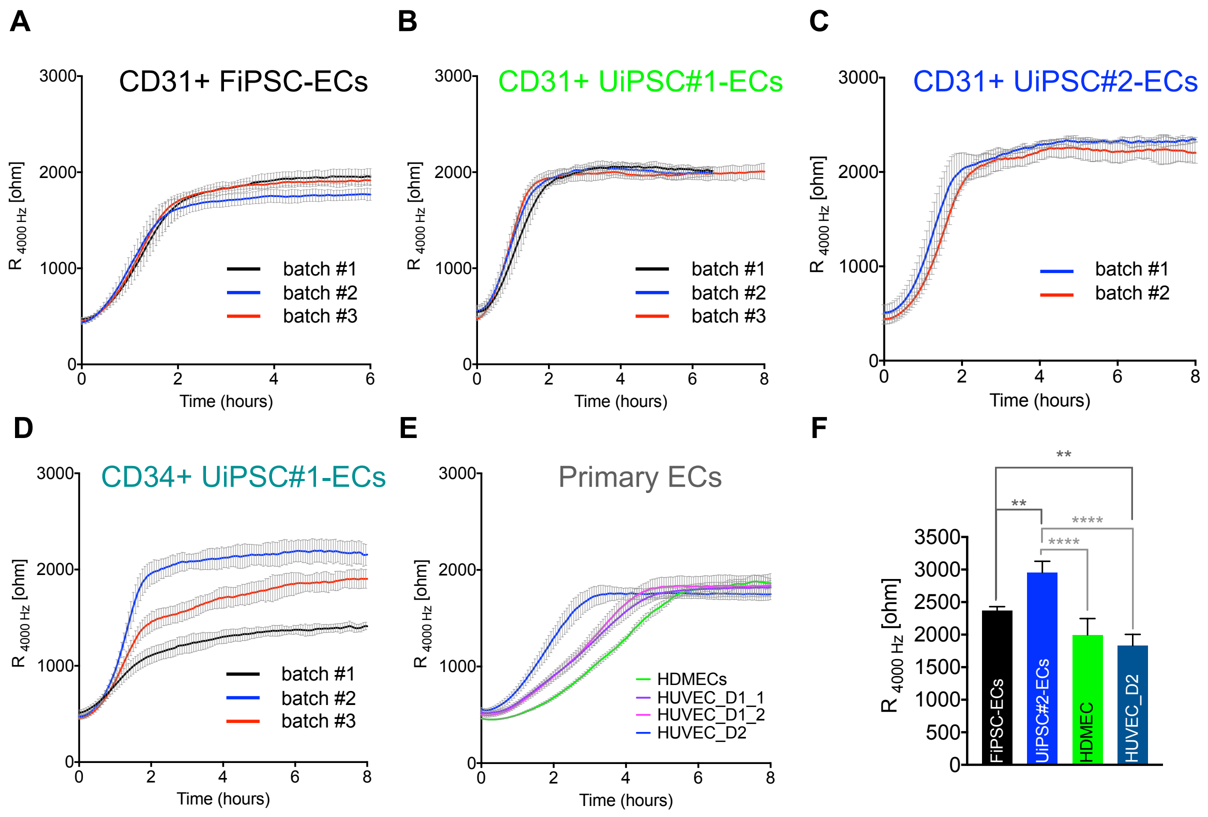

Figure S2. Related to Figure 2. Barrier properties of hiPSC-derived and primary ECs.
(A-E) Absolute resistance of independent batches of CD31+ and CD34+ hiPSC-derived and primary ECs: CD31+ FiPSC-ECs (A), CD31+ UiPSC#1-ECs (B), CD31+ UiPSC#2-ECs (C), CD34+ UiPSC#1-ECs (D), HDMECs and HUVECs (derived from two independent donors D1 and D2, and two independent isolations per donor D1_1 and D1_2) (E). Error bars are shown as ±SD of three to four independent wells. **(F)** Quantification of absolute resistance of CD31+ hiPSC-ECs and primary ECs (HDMECs and HUVECs from donor D2) from three independent biological experiments of the EC monolayer in EGM-2. Error bars are shown as ±SD of three independent biological experiments.

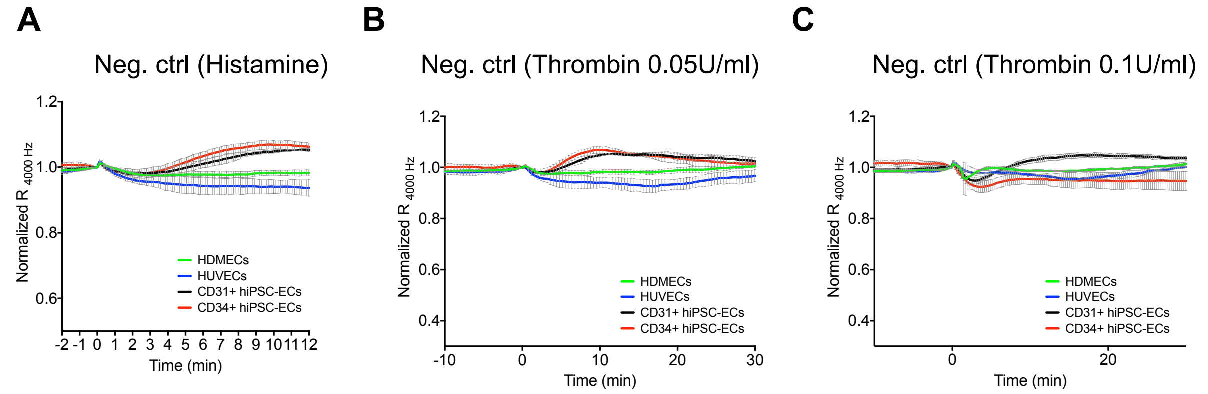

Figure S3. Related for Figure 3. Comparative assessment of barrier disruption in primary and hiPSC-derived ECs upon control (compound-free) treatment.
(A-C) Changes in normalized resistance at 4000 Hz of the endothelial monolayer upon control stimulation with equal volume of medium without the compound is shown. Normalized resistance is shown as a representative plot of one independent biological experiment. Error bars are shown ±SD of three to four independent wells.

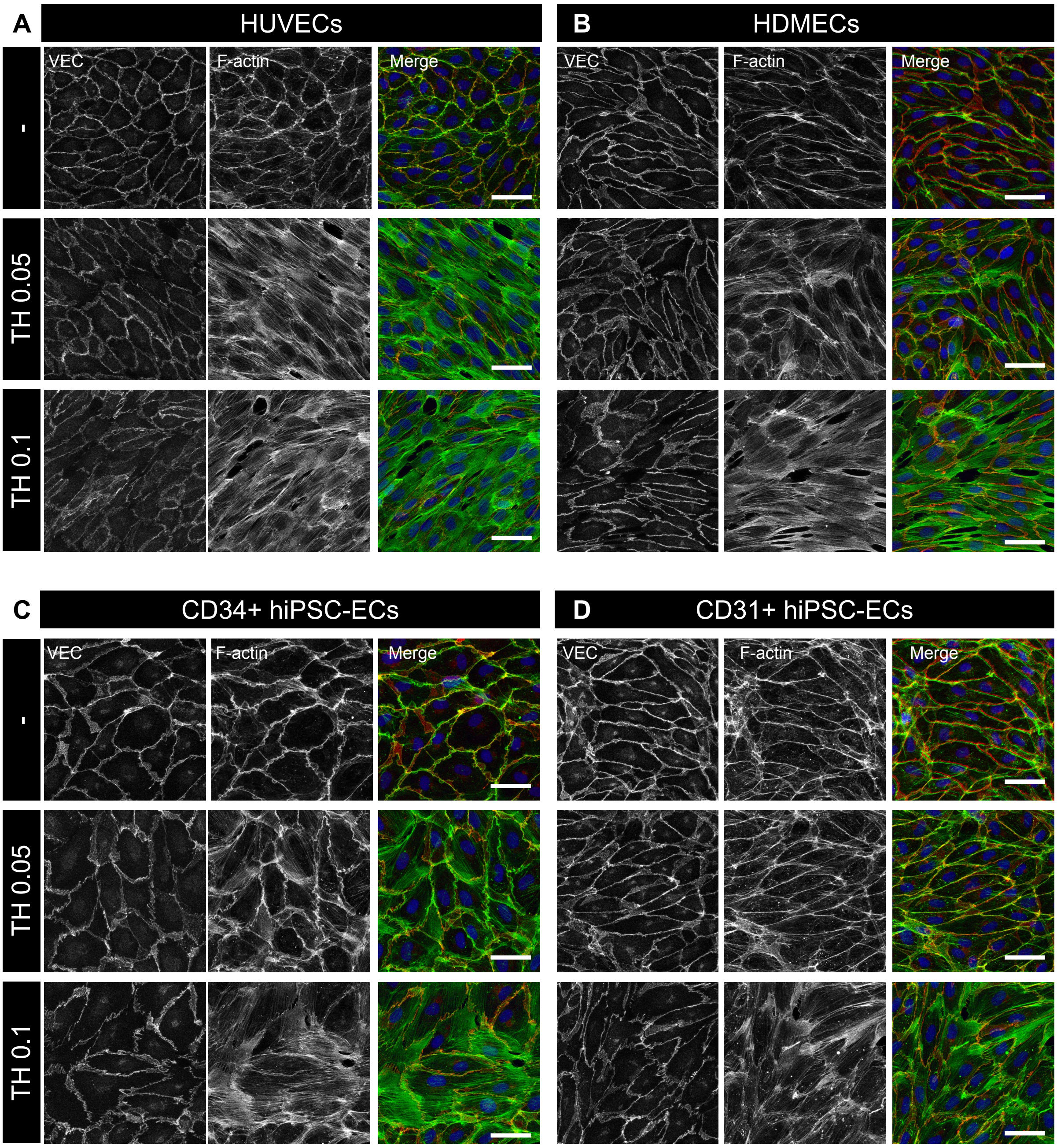

Figure S4. Comparison of junctional integrity in primary and hiPSC-ECs.
(A-D) Junctional integrity in primary cells and hiPSC-ECs was analysed using adherens junctional marker (VEC) counterstained with F-actin in HUVECs (A), HDMECs (B), CD34+ hiPSC-ECs (C) and CD31+ hiPSC-ECs (D) upon control stimulation with medium only (-) or thrombin (0.05U/ml and 0.1U/ml) for 30min. Disassembly of cell junctions and reorganisation of cortical actin and actin stress fibres formation can be observed in HUVECs and HDMECs upon thrombin (0.05U/ml and 0.1U/ml) stimulation. CD34+ hiPSC-ECs and CD31+ hiPSC-ECs shown robust response upon thrombin (0.1U/ml) stimulation. Representative pictures are shown from experiments performed three batches of CD31+ and CD34+ hiPSC-ECs, HUVECs from three batches (two donors, and two independent batches for one of the donors), HDMECs a single donor. Scale bar 50μm.

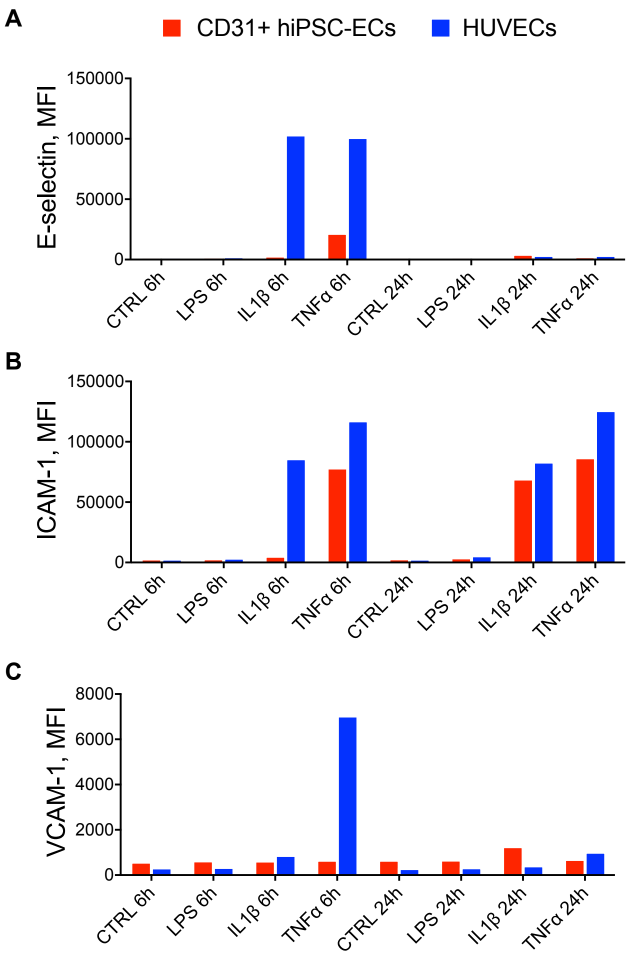

Figure S5. Related to Figure 5. Assessment of inflammatory responses in primary and hiPSC-ECs. (A-C) FACs analysis of surface expression of E-selectin (A), ICAM-1 (B) and VCAM-1 (C) after 6h and 24h post-treatment with LPS (100ng/ml), IL1β (10ng/ml) and TNFα (10ng/ml).

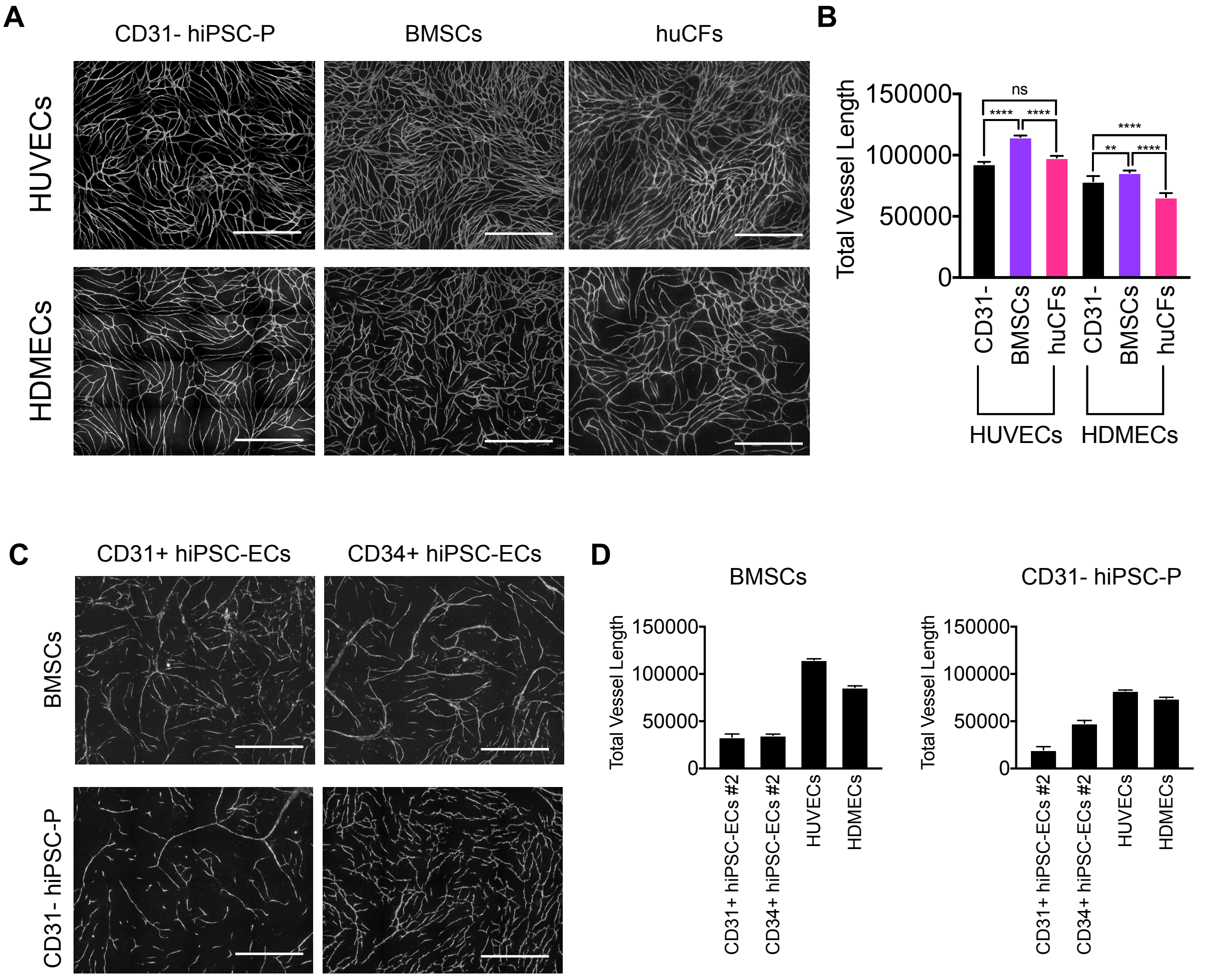

Figure S6. Related to Figure 6. Comparison the effect of different stroma cells in an *in vitro* vasculogenesis assay. (A) Representative immunofluorescent images of an *in vitro* vasculogenesis sprouting assay at day 10 of the co-culture of primary ECs (HUVECs and HDMECs) and different stroma cells (CD31- hiPSC-P, BMSCs and huCFs) used for quantification of the sprouting network. ECs are visualized with anti-CD31 (white). Automatically stitched images (10X objective, 4X4 focus planes) are shown. The images were taken with an automated imaging system with autofocus on CD31. **(B)** Quantification of EC sprouting network at day10 of the co-culture. Quantification was performed with Angiotool software. The total vessel length and total number of junctions are shown. Automatically stitched images (10X objective, 4X4 focus planes) from four to five co-cultures were used for quantification. Data are shown as ±SD. **(C)** Representative immunofluorescent images of an *in vitro* vasculogenesis sprouting assay at day 10 of the co-culture of CD31+ and CD34+ hiPSC-ECs with BMSCs and CD31- hiPSC-P used for quantification of the sprouting network. ECs are visualized with anti-CD31 (white). Automatically stitched images (10X objective, 4X4 focus planes) are shown. The images were taken with an automated imaging system with autofocus on CD31. **(D)** Quantification of EC sprouting network at day10 of the co-culture. Quantification was performed with Angiotool software. The total vessel length and total number of junctions are shown. Automatically stitched images (10X objective, 4X4 focus planes) from five co-cultures were used for quantification. Scale bar 1000μm. Data are shown as ±SD.

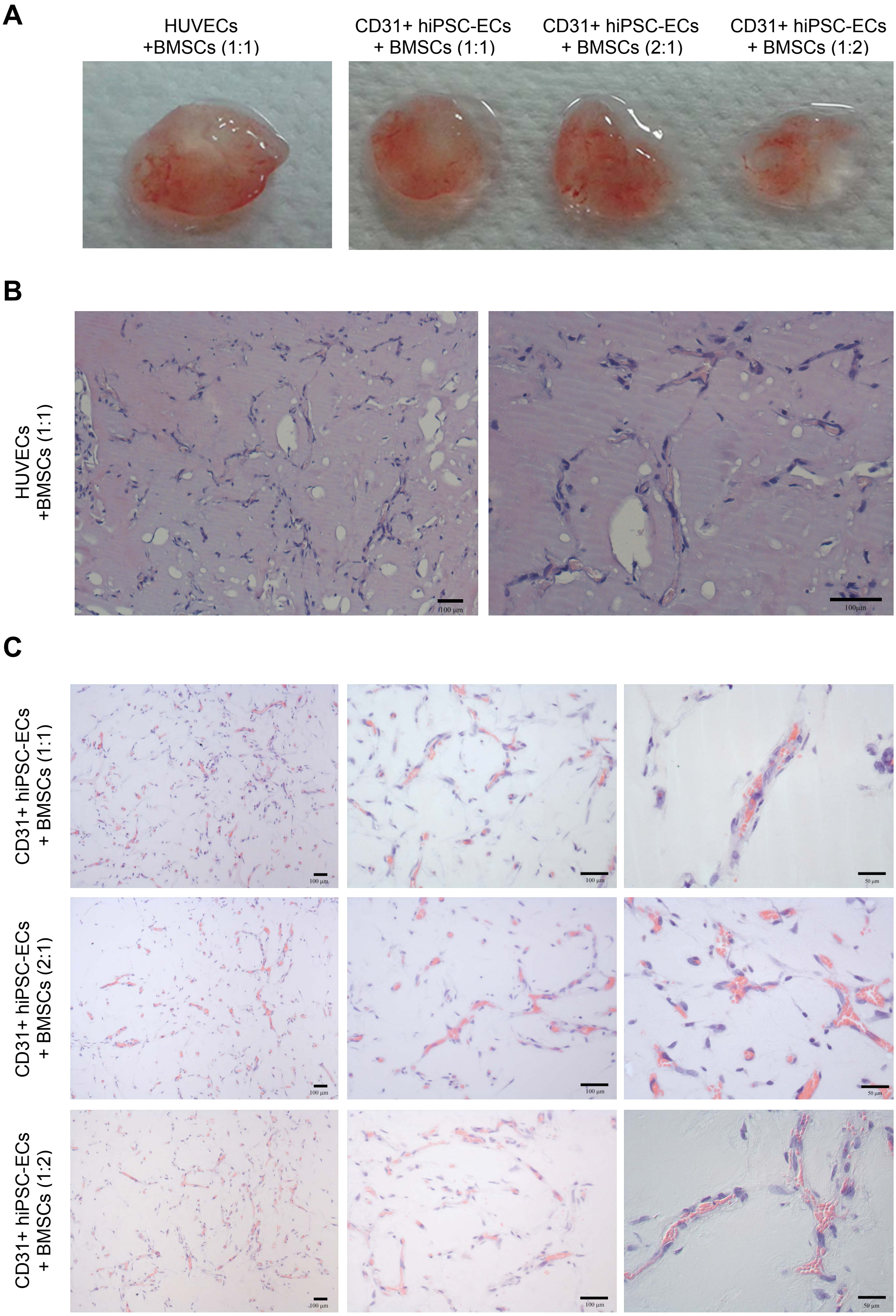

Figure S7. Related to Figure 7. Comparison of primary and hiPSC-ECs in an *in vivo* vasculogenesis assay. (A) Representative pictures of Martigel plugs 3 weeks post transplantation. **(B)** H&E images of Matrigel plugs. Representative images of Matrigel plugs with HUVECs and BMSCs (1:1). Scale bar 100μm. **(C)** Representative H&E images of Matrigel plugs with different ratios of hiPSC-ECs and BMSCs. Scale bar 100μm and 50μm.

Supplemental Tables

**Supplemental Table 1.** Sequence of primes used for qPCR			
**Gene**	**Forward sequence**	**Reverse sequence**	**Product size**
hARP	CACCATTGAAATCCTGAGTGATGT	TGACCAGCCCAAAGGAGAAG	116
VEC	GGCATCATCAAGCCCATGAA	TCATGTATCGGAGGTCGATGGT	100
VEGFR2	CCATCTCAATGTGGTCAACCTTCT	TCCTCAGGTAAGTGGACAGGTTTC	107
VEGFR3	CTGTGCCTGCGACTGTG	GGTGTCGATGACGTGTGACT	111
NOTCH1	ATAGTCTGCCACGCCTCTG	AGTGTGAAGCGGCCAATG	148
NOTCH4	GTGGTCATGGGTGTGGATT	CAGCAAGGAAGCGGAGTAG	94
JAG1	ACTGTCAGGTTGAACGGTGTC	ATCGTGCTGCCTTTCAGTTT	92
DLL4	TATGTGTGCCAGCCAGATG	ATGACAGCCCGAAAGACAG	90
SOX17	CGAGTTGAGCAAGATGCTGG	TTGTAGTTGGGGTGGTCCTG	120
NRP1	AACACCAACCCCACAGATG	AAGTTGCAGGCTTGATTCG	82
CX40	AATCAGTGCCTGGAGAATGG	CGAACCTGGATGAAACCTTC	146
EPHRINB2	GAAGTACGAGCCCCACAGA	CCCAACGCAGAAATAAACG	91
EPHB4	GAAAAGGAAGTGCCCAACA	CTGGCAAGGGAGTCACACT	99
APLNR	TTCTGCAAGCTCAGCAGCTA	GGTGCGTAACACCATGACAG	207
COUPTFII	GCTTTCCACATGGGCTACAT	CAAGTGGAGAAGCTCAAGGC	117

Supplemental Movies

Supplemental Movie 1. Related to Figure 5. Leukocyte adhesion to TNFα treated hiPSC-ECs.

Supplemental Experimental Procedures

Differentiation of hiPSCs towards ECs

hiPSCs were maintained in mTeSR-1 or mTeSR-E8 and differentiated towards ECs using previously published protocols (Orlova et al., 2014a; 2014b). For mesoderm induction (day 0-3), either combination of BMP4 (30ng/ml), ActA (25ng/ml) and CHIR (1.5μM) or CHIR (8μM) in B(P)EL were used, with the cells plated on MT or VN-coated plates respectively. The cultures were refreshed with vascular specification medium comprised of VEGF (50ng/ml) and SB431542 (10μM) in B(P)EL at day 3, day 6, and day 9. CD34+ ECs were isolated at day 6 of differentiation using EasySep™ CD34 Human Cord Blood Isolation Kit II (SCT) according to manufacturer's custom protocol (Giacomelli et al., 2017a; 2017b). CD31+ ECs were isolated at day 10 of differentiation using CD31 Dynabeads (Thermo Fisher Scientific), as previously described (Orlova et al., 2014b; 2014a). CD34+ hiPSC-ECs were plated post-isolation at the seeding density ~8,000cells/cm2 on fibronectin (FN)-coated plates, and CD31+ hiPSC-ECs were plated post-isolation at the seeding density ~12,000cells/cm2 on gelatin-coated plates (Orlova et al., 2014b). hiPSC-ECs were expanded in complete EC growth medium comprised of Human Endothelial-SFM (EC-SFM) with 1% platelet poor serum, VEGF (30ng/ml) and bFGF (20ng/ml), as described previously (Orlova et al., 2014b; 2014a). The cells were expanded for additional 3-4 days post-isolation and cryopreserved using serum-free cryopreservation medium (CryoStor™CS10)(SCT). For characterization and functional assays CD34+ and CD31+ hiPSC-ECs were thawed and cultured on FN or gelatin-coated plates respectively in complete EC growth medium.

Assessment of hiPSC-ECs functionality in an *in vitro* vasculogenesis assay

The co-culture experiments with hiPSC-ECs or primary ECs and stromal cells were performed essentially as previously described (Orlova et al., 2014b; 2014a). The following stromal cells were used in this study: CD31- hiPSC-P, derived as described (Orlova et al., 2014b; 2014a); human BMSCs and human cardiac fibroblasts were purchased from Promocell and cultured in the medium recommended by the cell supplier according to the supplier’s protocol. The co-cultures were stopped at day 10 and post-fixed and stained with anti-CD31 (DAKO) and anti-SOX17 (R&D) antibodies. The co-cultures were imaged with the EVOS FL AUTO2 Imaging system (ThermoFischer Scientific) with the 10X Objective for quantifications with autofocus on CD31, and auto stitching 4X4 focus planes or 20X Objective for CD31 and SOX17 images. The co-cultures were quantified using publicly available software AngioTool (Zudaire et al., 2011).

Endothelial barrier function analysis

Endothelial barrier function analysis was performed using impedance-based cell monitoring using electric cell-substrate impedance sensing system (ECIS Zθ, Applied Biophysics). CD34+ and CD31+ hiPSC-ECs were seeded on FN-coated ECIS arrays each containing 8 wells with 10 gold electrodes per well (8W10E PET, Applied Biophysics). The cell seeding density was estimated ~50,000cells/cm^2. For barrier function and migration studies the cells were seeded for at least 24h in complete EC growth medium followed by 6h serum starvation step in EC-SFM. For the assessment of cell migration after serum starvation, the medium was changed to EC-SFM or EC-SFM supplemented with VEGF 75ng/ml, and electric wound (10 sec pulse of 5V at 60 kHz) was applied to the cells 1h after medium change. Recovery of the barrier was monitored in real time over 6-12h. Multiple frequency/time (MFT) mode was used for the real-time assessment of the barrier and monolayer confluence. To assess the disruption of the EC barrier upon administration of histamine or thrombin, the medium was first changed to EGM-2 followed by application of a new electrical wound (to replace “old” cells on the electrodes with the “new” cells), and recovery of the barrier was monitored for another 24h. The medium was changed to EBM-2 (basal medium) 1-2h prior to stimulation. Histamine or thrombin stimulation was performed at single frequency/time (SFT) mode at 4kHz by removal of 100μl of growth medium and adding first 100μl of EBM-2 (negative control) followed by ~30min recording, removal of 100μl growth medium and adding 100μl of 4X concentrated stock of histamine to final concentration 10μM followed by 1h recording and removal of 100μl growth medium and adding 100μl of 4X concentrated stock of thrombin in EBM-2 medium with the end concentration (0.05 and 0.1U/ml).

Assessment of junctional integrity

Analysis of junctional integrity was performed as previous described (Orlova et al., 2006) with some modifications. Briefly, ECs were seeded on FN-coated 96-well black imaging plates (Corning) at the seeding density ~10,000cells/well in EGM-2 (primary ECs) or complete EC-SFM (hiPSC-ECs) medium. 48h post-seeding ECs were serum-starved in 100μl EBM-2 medium followed by thrombin stimulation by adding 100μl of 2X concentrated stock of thrombin in EBM-2 medium with the end concentration

(0.05 and 0.1U/ml). Cells were fixed with 4% paraformaldehyde (PFA, Sigma), permeabilized with the 0.1%TX-100 and stained with anti-ZO1 (ThermoFisher) or VEC (CellSignaling) and counterstained with A488 conjugated Phalloidin (ThermoFisher). High magnification images were acquired with the WLL1 confocal microscope (Leica), using 40x DRY objective using 0.75 Zoom factor.

Stimulation with pro-inflammatory cytokines

ECs were stimulated in complete EC growth medium with pro-inflmmatory cytokines (TNFα 10ng/ml, IL1β 10ng/ml) or LPS (100ng/ml) in complete EC growth medium. FACs analysis of the expression of E-selectin, ICAM-1 and VCAM-1 was performed at 6, 12 and 24h post-stimulation.

Flow adhesion assay for leukocyte-endothelial cell interaction

Vena8 Endothelia+ chips (Cellix) were coated with FN (50μg/ml) overnight (ON) at 4°C in a humidified chamber by injecting ~10μl into the microfluidic channel. ECs were stimulated in complete EC growth medium with TNFα (10ng/ml) for ~12h (ON). Next day ECs were detached, counted and re-suspended in EGM-2 medium (HUVECs) or complete EC-SFM (hiPSC-ECs) at ~1.5E6 cells/100μl. ECs were seeded into a microfluidic chip by injecting ~6μl of the cell suspension into the microfluidic channel. Microfluidic chips were incubated at 37°C for ~15min in a humidified chamber in order to facilitate cell attachment, and additional 40μl of medium was added from the both sides of the channel. The cells were incubated for 1h at 37°C, after 1h ~50-80μl of the medium was added from both sides of the channel and the chips were kept at 37°C prior to the assay. The assay was performed within ~2h of cell seeding. Human leukocytes (THP1) were washed once with PBS and re-suspend in 1ml of PBS with DiOC6 (1:5000)(Sigma). THP1 cells were incubated in the dark at RT for 10min at RT, washed with PBS and re-suspend in complete RPMI medium at the end concentration 2.5E6 cells/ml. THP1 cells for flow adhesion experiments were perfused for 5 minutes at 0.5 dyne/cm2, followed by a 5 min wash with RPMI medium. The number of adherent fluorescently labelled THP1 cells on ECs was quantified using CellProfiler (Carpenter et al., 2006).

Assessment of hiPSC-ECs functionality in an *in vitro* vasculogenesis assay

The co-culture experiments with hiPSC-ECs or primary ECs and stromal cells were performed essentially as previously described (Orlova et al., 2014b; 2014a). Details are provided in supplemental experimental procedures.

Transplantation of hiPSC-ECs and BMSCs

The Matrigel plug assay using hiPSC-ECs and BMSCs was performed as previously described (Sacchetti et al., 2016). Experiments were carried out in compliance with relevant Italian laws and Institutional guidelines and all procedures were IACUC approved. hiPSC-ECs and BMSCs were suspended in 1 ml GF-reduced Matrigel (BD Biosciences Labware) at different ratios: 1 million hiPSC-ECs and one million BMSCs (1:1), 2 million hiPSC-ECs and 1 million BMSCs (2:1), 1 million hiPSC-ECs and 2 million BMSCs (1:2). In control samples, 1 million HUVECs were mixed with 1 million BMSCs. Cell suspensions (~ 0.7 ml) were injected subcutaneously in the back of SCID/beige mice (CB17.Cg-Prkdcscid Lyst bg-J/Crl; Charles River). Three weeks after transplantation, the plugs were harvested, fixed in 4% neutral buffered formaldehyde for 24hr at 4°C and routinely embedded in paraffin. Five-micron-thick sections were cut from paraffin blocks for hematyoxilin and eosin (H&E) staining and immunohistochemistry. For human CD31 immunolocalization, deparaffinized sections were incubated with mouse anti-Human CD31 antiserum (Endothelial Cell Clone JC70A; M0823 Dako) diluted 1:30 in phosphate buffered saline (PBS) for 2h at RT, washed with PBS and then exposed for 30 min' at RT to Alexafluor goat anti Mouse IgG1 488 (A-21121; ThermoFisher Scientific) diluted 1:200 in PBS. For DNA counterstaining, sections were incubated for 15 min at RT with Topro-3 (T3605 ThermoFisher Scientific) diluted1:1000 in PBS.

Transplantation of hiPSC-ECs and CD31- hiPSC-P

Matrigel plug assay with hiPSC-ECs and CD31- hiPSC-P was performed similar to BMSCs transplantation experiments with minor modifications. Animal experiments were approved by the Leiden University Medical Centre animal experimental committee and the Commission Biotechnology in Animals of the Dutch Ministry of Agriculture. Two million hiPSC-ECs and one million CD31- hiPSC-P were mixed in 600 µl of GF-reduced Matrigel (BD Biosciences Labware), supplemented with bFGF (1ug/ml), VEGF (200ng/ml) and Heparin (2.5U) and injected subcutaneously in the back region of 8-10 weeks old male NSG mice (NOD.Cg-Prkdc^{scid} Il2rg^{tm1Wjl}/SzJ, Charles River). Three mice were used per group. After cell injection, each mouse received subcutaneous injections of 100ng/μl bFGF every 48

hours. Three weeks after transplantation, the plugs were harvested, and immersed in 4% formaldehyde in Phosphate-Buffered solution at RT for 4h, followed 15% Sucrose-PBS solution for a minimum of 2h and subsequently to 30% Sucrose-PBS solution O/N at 4°C. Plugs were embedded in Tissue-Tek® OCT compound (Sakura® Finetek) for further analysis. Eight-micron-thick frozen sections were processed for H&E. For immunofluorescence staining sections were fixed with PBS/2% formaldehyde for 10 minutes at RT, permeabilized for 8 minutes with PBS/0.1% Triton-X-100 and incubated with the primary antibody were at 4°C O/N, followed by the secondary antibody for 1 hour at RT (for antibodies see Table S1). DNA counterstaining was performed with DAPI (1:1000, company). To quantify labelling for mouse and human specific fluorescence, labelled sections were digitalized using the Pannoramic Viewer software (3DHistech). For each plug 3 to 4 sections at 80-100 um distance were analyzed. In total 11 pictures per plug (20X magnification) were analyzed. The vascular density of human CD31 positive cells and CD31 mouse and human positive cells was measured by determining the area percentage above threshold on a large representative set of images. The threshold was determined interactively and was kept constant for the complete set. The analysis was performed using an in-house image analysis package, called Stacks.

Supplemental References

Carpenter, A.E., Jones, T.R., Lamprecht, M.R., Clarke, C., Kang, I.H., Friman, O., Guertin, D.A., Chang, J.H., Lindquist, R.A., Moffat, J., Golland, P., Sabatini, D.M., 2006. CellProfiler: image analysis software for identifying and quantifying cell phenotypes. Genome Biology 7, R100. doi:10.1186/gb-2006-7-10-r100

Giacomelli, E., Bellin, M., Orlova, V.V., Mummery, C.L., 2017a. Co-Differentiation of Human Pluripotent Stem Cells-Derived Cardiomyocytes and Endothelial Cells from Cardiac Mesoderm Provides a Three-Dimensional Model of Cardiac Microtissue. Curr Protoc Hum Genet 95, 21.9.1–21.9.22. doi:10.1002/cphg.46

Giacomelli, E., Bellin, M., Sala, L., van Meer, B.J., Tertoolen, L.G.J., Orlova, V.V., Mummery, C.L., 2017b. Three-dimensional cardiac microtissues composed of cardiomyocytes and endothelial cells co-differentiated from human pluripotent stem cells. Development dev.143438–47. doi:10.1242/dev.143438

Orlova, V.V., Drabsch, Y., Freund, C., Petrus-Reurer, S., van den Hil, F.E., Muenthaisong, S., Dijke, P.T., Mummery, C.L., 2014a. Functionality of endothelial cells and pericytes from human pluripotent stem cells demonstrated in cultured vascular plexus and zebrafish xenografts. Arteriosclerosis, Thrombosis, and Vascular Biology 34, 177–186. doi:10.1161/ATVBAHA.113.302598

Orlova, V.V., Economopoulou, M., Lupu, F., Santoso, S., Chavakis, T., 2006. Junctional adhesion molecule-C regulates vascular endothelial permeability by modulating VE-cadherin-mediated cell-cell contacts. Journal of Experimental Medicine 203, 2703–2714. doi:10.1084/jem.20051730

Orlova, V.V., van den Hil, F.E., Petrus-Reurer, S., Drabsch, Y., Dijke, ten, P., Mummery, C.L., 2014b. Generation, expansion and functional analysis of endothelial cells and pericytes derived from human pluripotent stem cells. Nature Protocols 9, 1514–1531. doi:10.1038/nprot.2014.102

Sacchetti, B., Funari, A., Remoli, C., Giannicola, G., Kogler, G., Liedtke, S., Cossu, G., Serafini, M., Sampaolesi, M., Tagliafico, E., Tenedini, E., Saggio, I., Robey, P.G., Riminucci, M., Bianco, P., 2016. No Identical “Mesenchymal Stem Cells” at Different Times and Sites: Human Committed Progenitors of Distinct Origin and Differentiation Potential Are Incorporated as Adventitial Cells in Microvessels. STEMCR 6, 897–913. doi:10.1016/j.stemcr.2016.05.011

Zudaire, E., Gambardella, L., Kurcz, C., Vermeren, S., 2011. A Computational Tool for Quantitative Analysis of Vascular Networks. PLoS ONE 6, e27385–12. doi:10.1371/journal.pone.0027385
